# Interleukin-33 is activated by allergen- and necrosis-associated proteolytic activities to regulate its alarmin activity during epithelial damage

**DOI:** 10.1038/s41598-018-21589-2

**Published:** 2018-02-20

**Authors:** Ian C. Scott, Jayesh B. Majithiya, Caroline Sanden, Peter Thornton, Philip N. Sanders, Tom Moore, Molly Guscott, Dominic J. Corkill, Jonas S. Erjefält, E. Suzanne Cohen

**Affiliations:** 1 Department of Respiratory, Inflammation and Autoimmunity, MedImmune, Granta Park, Cambridge, CB21 6GH United Kingdom; 2 Neuroscience, Innovative Medicines and Early Development Biotech Unit, AstraZeneca, Granta Park, Cambridge, CB21 6GH United Kingdom; 3 Department of Experimental Medical Sciences, Lund University, Lund, Sweden

## Abstract

Interleukin (IL)-33 is an IL-1 family alarmin released from damaged epithelial and endothelial barriers to elicit immune responses and allergic inflammation via its receptor ST2. Serine proteases released from neutrophils, mast cells and cytotoxic lymphocytes have been proposed to process the N-terminus of IL-33 to enhance its activity. Here we report that processing of full length IL-33 can occur in mice deficient in these immune cell protease activities. We sought alternative mechanisms for the proteolytic activation of IL-33 and discovered that exogenous allergen proteases and endogenous calpains, from damaged airway epithelial cells, can process full length IL-33 and increase its alarmin activity up to ~60-fold. Processed forms of IL-33 of apparent molecular weights ~18, 20, 22 and 23 kDa, were detected in human lungs consistent with some, but not all, proposed processing sites. Furthermore, allergen proteases degraded processed forms of IL-33 after cysteine residue oxidation. We suggest that IL-33 can sense the proteolytic and oxidative microenvironment during tissue injury that facilitate its rapid activation and inactivation to regulate the duration of its alarmin function.

## Introduction

Interleukin (IL)-33 is a constitutively expressed IL-1 family cytokine alarmin predominantly localised in the nucleus of epithelial cells in barrier tissues and in endothelial cells in blood vessels. IL-33, like other IL-1 family cytokines, plays a major role in the initiation and amplification of immune responses and deregulated activity of these cytokines can lead to inflammatory, infectious and autoimmune diseases^1–3^. IL-33 is rapidly released from cells during necrosis or tissue injury and signals through a cell surface receptor complex of ST2 (IL-1 receptor-like 1, IL1RL1) and IL-1 receptor accessory protein (IL1RAcP) to initiate inflammatory pathways in immune cells such as type-2 innate lymphoid cells (ILC2), mast cells and natural killer (NK) cells^4–6^.

Although advances have been made into the physiological and pathological roles of IL-33, mechanisms regulating its alarmin activity remain poorly understood. IL-33 is produced as a full length (FL) protein containing 270 amino acids (aa) in human and 266 aa in mice. The N-terminus (1–75 aa) contains a nuclear localization sequence, a homeodomain-like helix-turn-helix DNA-binding domain and a chromatin binding domain^7^. IL-33 does not contain a signal sequence and its release mechanisms are unclear but release can occur by mechanical and oxidative stress, necrotic cell death, or cell activation through ATP signalling in the absence of cell death^8–11^. Genetic deletion of the N-terminal domain of IL-33 resulted in elevated levels of mature IL-33 in the serum and lethal ST2-dependent inflammation, demonstrating the key role of this region in regulating IL-33 release and activity^12^.

FL IL-33 has modest biological activity that can be enhanced by removal of the N-terminus^13–15^ or terminated by cleavage within the IL-1-like domain by caspases during apoptotic cell death^8,10,16^. Conversely, processed forms of IL-33 can be rapidly inactivated by disulphide bonding (DSB) of critical cysteine residues^17^. Despite these observations, a greater understanding of the mechanisms of proteolytic activation and inactivation of IL-33 *in vivo* and how this interacts with its release and oxidation is required.

Serine proteases from neutrophils (cathepsin G (CG), neutrophil elastase (NE) and proteinase-3 (PR-3)), mast cells (chymase and tryptase), and cytotoxic lymphocytes (granzyme B (gzmB)) are proposed to N-terminal process IL-33 into mature forms with up to 30-fold more potent activity^13–15^. *In vitro* studies have also suggested that IL-33 might be processed by calpain however the cleavage site and biological roles remain unclear^18^. In this study we utilised dipeptidyl peptidase I (DPP-1, Cathepsin C) deficient mice (*Ctsc*^−/−^), which lack NE, CG, PR-3, gzmB and chymase activities and have a 75% reduction in tryptase activity^19–21^, to investigate potential roles of DPP-1 activated immune cell proteases in IL-33 processing *in vivo*.

Mouse lungs challenged with the fungal allergen *Alternaria alternata* (ALT)^9,22^ induces the rapid release of an ~18 kDa form of IL-33 in bronchioalveolar lavage (BAL)^17^ consistent with an NE/CG processing site after residue Phe 101^15^. Here we challenged the lungs of *Ctsc*^−/−^ and wild type (WT) mice with ALT, however, in contrast to expectations, we found IL-33 processing can occur independently of DPP-1-activated immune cell proteases. Instead, we show that allergen and calpain proteases can proteolytically activate IL-33. In addition, allergen proteases can degrade mature DSB-IL-33. We show that multiple mature forms of IL-33 are present in human lung consistent with some, but not all, proposed processing sites. Thus, we report new mechanisms whereby IL-33 can sense the local microenvironment during epithelial tissue damage to enhance and also limit its alarmin activity.

## Results

### IL-33 processing in ALT-challenged mice can occur independently of immune cell proteases

**Figure 1:**
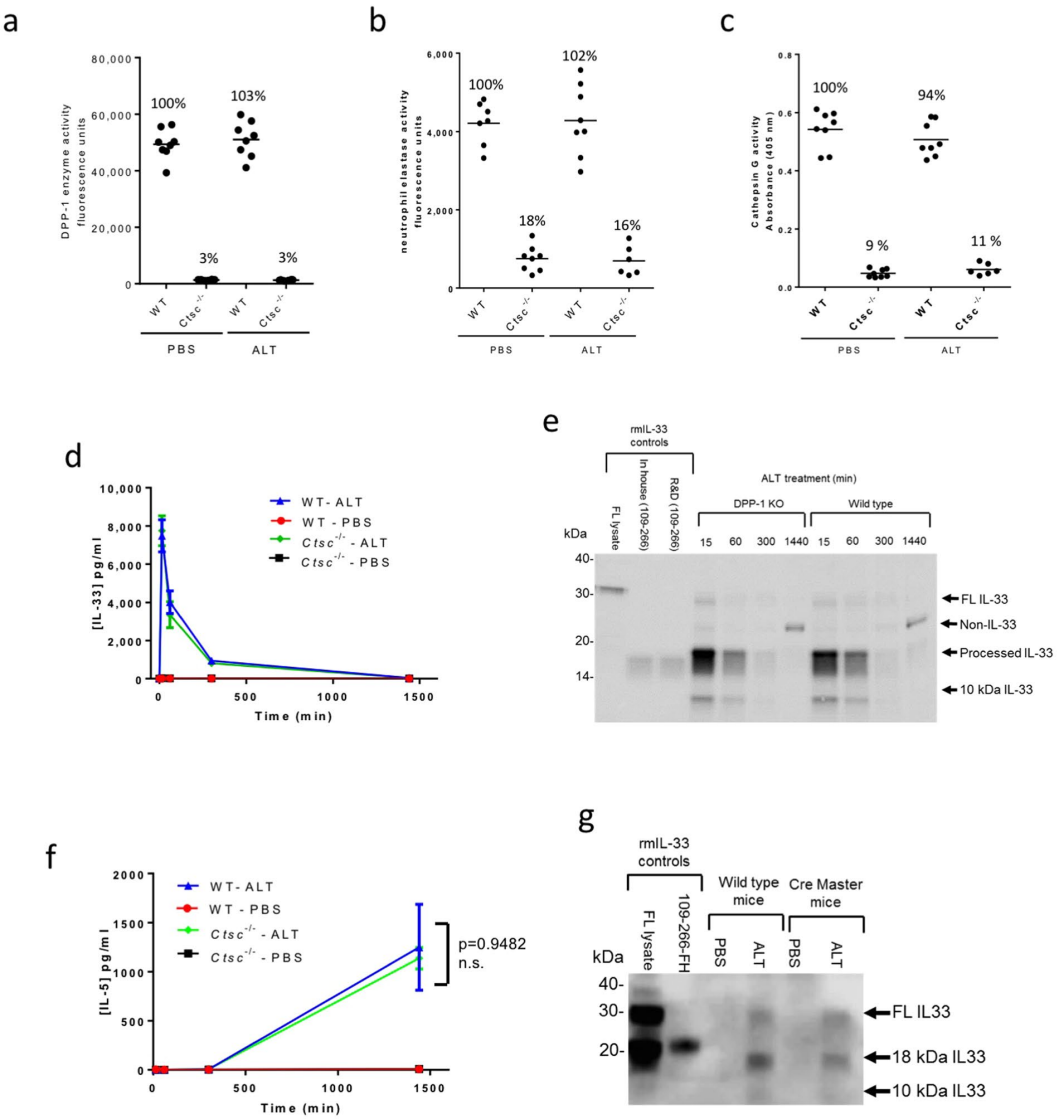
IL-33 processing in ALT model is independent of immune cell proteases. DPP-1 (**a**), NE (**b**), and CG (**c**) enzyme activities in bone marrow (BM) lysates from wild type (WT) and *Ctsc*^−/−^ mice 30 min after ALT or PBS challenge (n = 6–8/mice group). %Activity (relative to WT/PBS group) is shown above the data. (**d**) Concentration of IL-33 (pg/ml) in BAL from WT and *Ctsc*^−/−^ mice 15 min to 24 h after i.n. ALT or PBS challenge measured by ELISA (n = 3–4 mice/group). Data points are mean ± SEM. (**e**) Western blot of IL-33 of BAL samples (n = 3–4 pooled /group). Controls are as follows: FL lysate, lysate of CHO cells transfected with full length mouse IL-33; in house (109–266), in house generated recombinant mouse IL-33; mouse R&D (109–266), R&D systems recombinant mouse IL-33 (109–266 aa). (**f**) Concentration of IL-5 (pg/ml) in BAL from WT and *Ctsc*^−/−^ mice 15–1440 min after i.n. ALT or PBS challenge (n = 3–4 mice/group). Data points are mean ± SEM. Statistical analysis: two-way ANOVA test, Tukey’s post-test, F = 7.4, degrees of freedom = 9. n.s.: non-significant between WT/ALT and *Ctsc*^−/−^/ALT groups at 1440 min. (**g**) Western blot of IL-33 in BAL samples (n = 3–4 pooled /group) from WT and mast cell-deficient mice 30 min after ALT or PBS challenge. Controls: FL lysate, lysate of CHO cells transfected with full length mouse IL-33; 109–266-FH, recombinant mouse IL-33 109–266 aa with N-terminal Flag-His tag. Data is pooled from n = 3 independent studies (**a–c**). Data representative of n = 3 (**d–f**) and n = 2 (**g**) independent studies. Western blot images (**e** and **g**) have been cropped for clarity with full blots presented in Fig. S6.

To investigate the role of DPP-1-activated immune cell proteases in IL-33 processing *in vivo* we challenged the lungs of *Ctsc*^−/−^ and wild type (WT) mice with the *Alternaria alternata* (ALT) extract to induce IL-33 release and processing. However, despite reductions in DPP-1, NE and CG activity in *Ctsc*^−/−^ mice (Fig. 1a–c), the magnitude and time course of IL-33 release into BAL after ALT challenge (Figs 1d, S1a) and ratio of FL to ~18 kDa forms of IL-33 (Figs 1e, S1b, S6**)** were similar in WT and *Ctsc*^−/−^ mice. Importantly, low levels of protease activities in NE and CG assays in *Ctsc*^−/−^ mice are attributable to non-specific protease cleavage of the peptide substrates and not residual NE or CG activities^19^. Consistent with this lack of IL-33 modulation, IL-5 production, previously shown to be IL-33 dependent in this model^9^, was not significantly different between WT and *Ctsc*^−/−^ mice (Fig. 1f). That N-terminal processing of IL-33 occurred mainly independent of mast cells was confirmed in mast cell-deficient (Cre-master) mice^23^ where only marginally reduced processing was observed (Figs 1g, S6).

Collectively these results strongly suggest that IL-33 processing in ALT challenged mice can occur largely independently of DPP-1-activated and mast cell proteases previously implicated in IL-33 processing and suggest that alternative processing mechanisms exist.

### Rapid N-terminal processing and release of IL-33 occurs during early damage to airway epithelium

**Figure 2:**
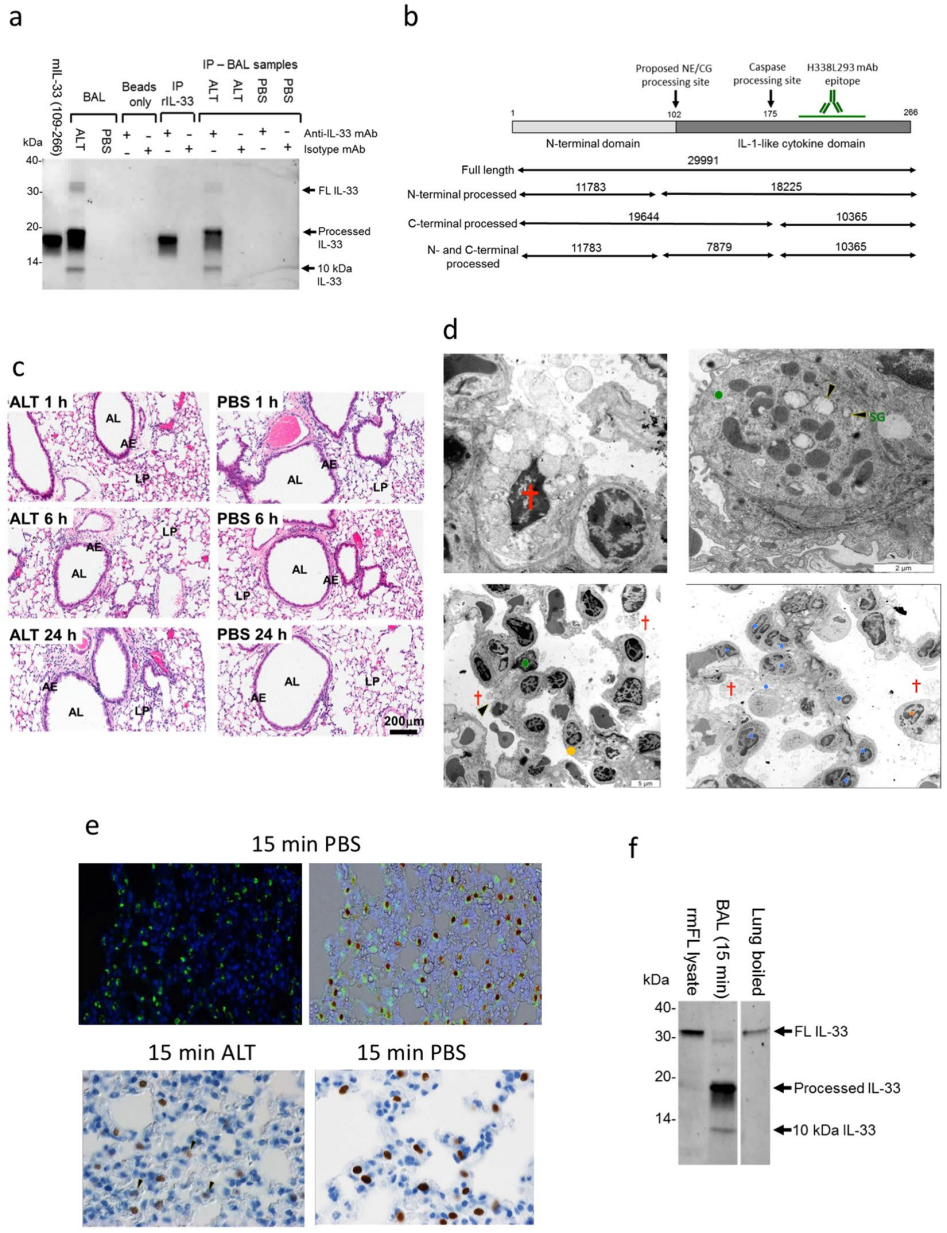
ALT drives rapid N-terminal processing and release of IL-33 from damaged airway epithelium. (**a**) Immunoprecipitation (#H338L293 or NIP228 mAb) and western blot (AF3626 mAb) of IL-33 in BAL (pooled n = 3–4 mice/group) after ALT or PBS challenge. Controls: mIL-33 (109–266), recombinant mouse IL-33 (109–266 aa). Abbreviations: IP, immunoprecipitation. (**b**) Diagram of the mouse IL-33 structure, proposed CG/NE and caspase sites, epitope for H338L293 mAb and theoretical MW (Da) of proteins. (**c**) Haematoxylin and eosin staining of mouse lung sections 1, 6 and 24 h after i.n. 25μg ALT or PBS challenge. AL, airway lumen; AE, airway epithelium; LP, lung parenchyma. Scale bar = 200 μm. (**d**) Transmission electron micrographs of mouse lung 15 min after ALT or PBS challenge. Top left: high power image of necrotic type 2 pneumocyte (red cross) 15 min after ALT challenge. Top right: high power image of intact type 2 pneumocyte (green dot) 15 min after PBS challenge. SG, surfactant granules (black arrow heads). Bottom left: low power image of necrotic type 2 pneumocytes 15 min after ALT challenge. Eosinophil granulocyte (orange dot). Bottom right: Low power image of neutrophil (blue dots) and eosinophil infiltration 15 min after ALT challenge. Scale bars 2 μm (high power images) and 5 μm (low power images). (**e**) Immunostaining of pro-surfactant protein C (green stain, upper left panel) and overlaid with IL-33 (brown stain, upper right panel) in mouse alveolar tissue 15 min after PBS challenge. Immunostaining of IL-33 in alveolar tissue 15 min after ALT (lower left panel) or PBS challenge (lower right panel). Fading of IL-33 staining is seen following ALT challenge (black arrow heads). (**f**) Western blot of IL-33 in BAL (pooled n = 3 mice) 15 min after ALT challenge and boiled mouse lung (pooled n = 3 mice). Controls: FL lysate, lysate of CHO cells transfected with full length mouse IL-33. Representative images from study with n = 3 mice/group (**a–c**). Representative of n = 3 independent studies (**d–f**). Western blot images (**a** and **f**) have been cropped for clarity with full blots presented in Fig. S7.

To understand further the results obtained with the *Ctsc*^−/−^ mice, we sought to determine if the ~18 kDa form of IL-33 in BAL from ALT challenged mice was indeed the result of N-terminal processing. Cleavage within the IL-1-like domain before residue Asp 178, which would generate IL-33 fragments of a similar size, has been previously reported^8,16^. Immunoprecipitation (IP) of IL-33 in BAL obtained 15 minutes after ALT challenge in mice using a C-terminal anti-IL-33 mAb (H338L293)^17^ detected a ~18 kDa form as well as FL and a ~10 kDa fragment of IL-33 (Figs 2a, S7). These data are consistent with significant N-terminal processing but also some additional cleavage within the IL-1-like domain of IL-33. A schematic diagram of mouse IL-33, proposed processing sites, epitope for H338L293 mAb and predicted MW of N- and C-terminal mature forms of mouse IL-33 are shown in Fig. 2b.

To help identify alternative pathways for IL-33 processing we further characterised the ALT model. ALT challenge did not change lung morphology when assessed by histological staining up to 24 h after challenge (Fig. 2c). However, transmission electron microscopy (TEM) of ALT challenged lungs, 15 min to 4 h after treatment, revealed rapid necrotic damage to the epithelial barrier, extracellular debris, and pronounced cytolysis of type 2 pneumocytes with dissolved cytoplasms and free-floating organelles (Fig. 2d top left panel). PBS treated lungs showed less evidence of damage with intact type 2 pneumocytes containing lamellar surfactant granules (Fig. 2d top right panel). TEM also revealed a low-grade immune cell infiltration, including neutrophils and eosinophils, 15 min after ALT challenge (Fig. 2d bottom left and right panels respectively). Neutrophils were largely confined to the vascular spaces within pulmonary capillaries and had not infiltrated into interstitial tissue (Fig. 2d bottom right panel). Substantial infiltration of immune cells into BAL was only detected 5–24 h after ALT challenge (Fig. S2a).

IL-33 was expressed in pro-surfactant protein C positive (pSPC^+^) type 2 pneumocytes in the alveolar region of mouse lungs (Fig. 2e, upper left and right panels) with IL-33 immunoreactivity reduced 15 min after ALT challenge (Fig. 2e, lower left and right panels). IL-33 was detected exclusively as a FL ~30 kDa protein in unchallenged, undamaged mouse lung (Figs 2f, S7). However, 15 min after ALT challenge IL-33 was released into BAL predominantly as a ~18 kDa mature form (Figs 2f, S7). IL-33 release and processing was co-incident with lactate dehydrogenase (LDH) release (Fig. S2b,c) consistent with cytolysis of type 2 pneumocytes.

Collectively these data are consistent with N-terminal processing of endogenous IL-33 occurring concurrent with its release during the early ALT-driven necrosis of type 2 pneumocytes, rather than latterly during the peak of ALT-driven immune cell infiltration.

### Calpain can process endogenous IL-33 in airway epithelial cells

**Figure 3:**
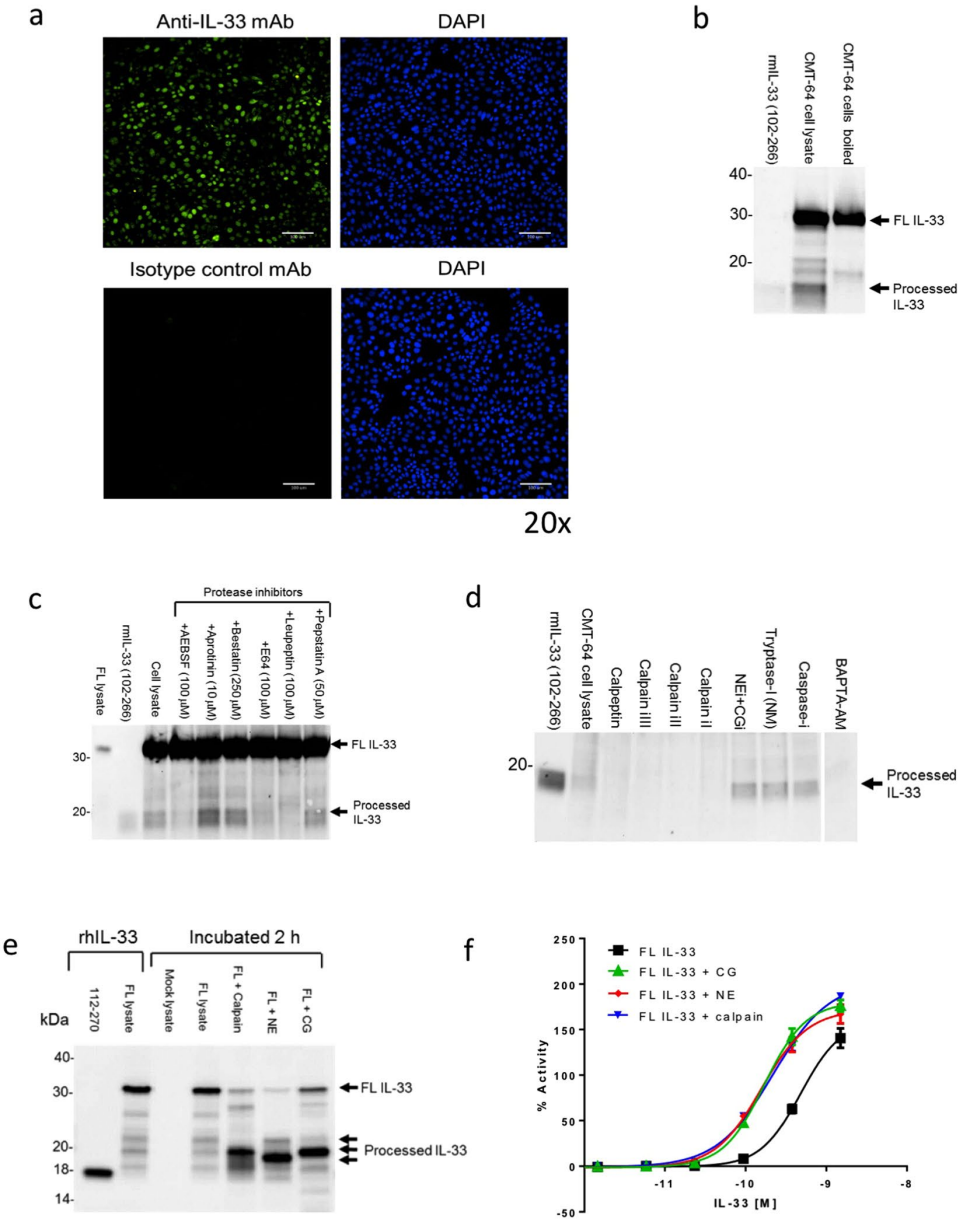
Calpain can process IL-33 in airway epithelial cells and enhance its activity. (**a**) Immunostaining of mouse IL-33 (top left panel), with isotype control antibodies (bottom left) and cell nuclei (right panels) in mouse CMT-64 cells at 20× magnification. (**b**) Western blot of mouse IL-33 in CMT-64 cell lysates (PBS, 0.1% Triton X100) and CMT-64 cells (boiled in SDS-PAGE buffer). Controls: rmIL-33 (102–266), recombinant mouse IL-33 (102–266 aa). (**c**) Western blot of mouse IL-33 in CMT-64 cell lysates. Cells were pre-treated for 30 min with protease inhibitors and incubated and in PBS/0.1% Triton X100 for 30 min. Controls: as (b). (**d**) Western blot of mouse IL-33 in CMT-64 cell lysates. Gel image is cropped to show only processed IL-33. Cells were pre-treated for 30 min with protease inhibitors, BAPTA-AM or 0.1% DMSO (cell lysate) and incubated for 30 min in PBS/0.1% Triton X100. Controls as (b). Abbreviations: i, inhibitor, NM, nafamostat mesylate. (**e**) Western blot of FL human IL-33 lysate incubated alone or with calpain-1, NE and CG for 2 h. Controls: rhIL-33, recombinant human IL-33, 112–270, purified recombinant human IL-33 (112–270 aa); FL lysate, lysate of HEK cells transfected with full length human IL-33; Mock lysate, lysate of mock transfected HEK cells. (**f**) Relative NFkB p65/RelA translocation in HUVECs 30 min after stimulation with human FL IL-33 lysate and FL IL-33 pre-incubated with calpain-1, NE or CG. Data points are mean ± SEM. of duplicate determinations. %Activity is calculated relative to signal of 3 ng/ml rhIL-33 (112–270 aa). Representative of n = 3 independent experiments (**a–f**). Western blot images (**b–e**) have been cropped for clarity with full blots presented in Figs S8–S9.

We hypothesized that endogenous proteases from damaged airway epithelial cells might be responsible for rapid processing of IL-33 following ALT challenge. CMT-64, a mouse alveolar type 2 pneumocyte-like epithelial cell line^24^ contained high levels of FL IL-33 (Fig. 3a) that was processed to an ~18 kDa form upon cell damage with detergent (Figs 3b, S8). Alternatively, treatment of CMT-64 with the calcium ionophore, ionomycin, increased release of FL and ~18 kDa IL-33 (Fig. S3a and b) concurrent with cell damage (Fig. S3c). Broad-spectrum protease inhibitors implicated cysteine protease activity in the processing of IL-33 in CMT-64 cells (Figs 3c, S8) and indeed calpain inhibitors (calpeptin, calpain inhibitors-I, -II and –III^25,26^), as well as the calcium chelator BAPTA-AM^27^, completely inhibited the generation of ~18 kDa IL-33 (Figs 3d, S3d and S9). Processing of endogenous IL-33 in primary normal human bronchial epithelial cells (NHBE) was also dependent on calpain activity (Fig. S3e).

### Calpain-1 processes IL-33 to enhance its alarmin activity

Although Hayakawa *et al*. have previously shown IL-33 cleavage by calpains^18^ the functional consequence of calpain cleavage of IL-33 have not been previously addressed. *In vitro*, calpain-1 processed FL hIL-33 to a ~20 kDa form (Figs 3e, S9) and enhanced its activity ~3-fold, comparable with CG and NE (Fig. 3f). In contrast, mature hIL-33 (112–270 aa) was not altered by calpain-1, CG and NE (Fig. S3f,g).

**Figure 4:**
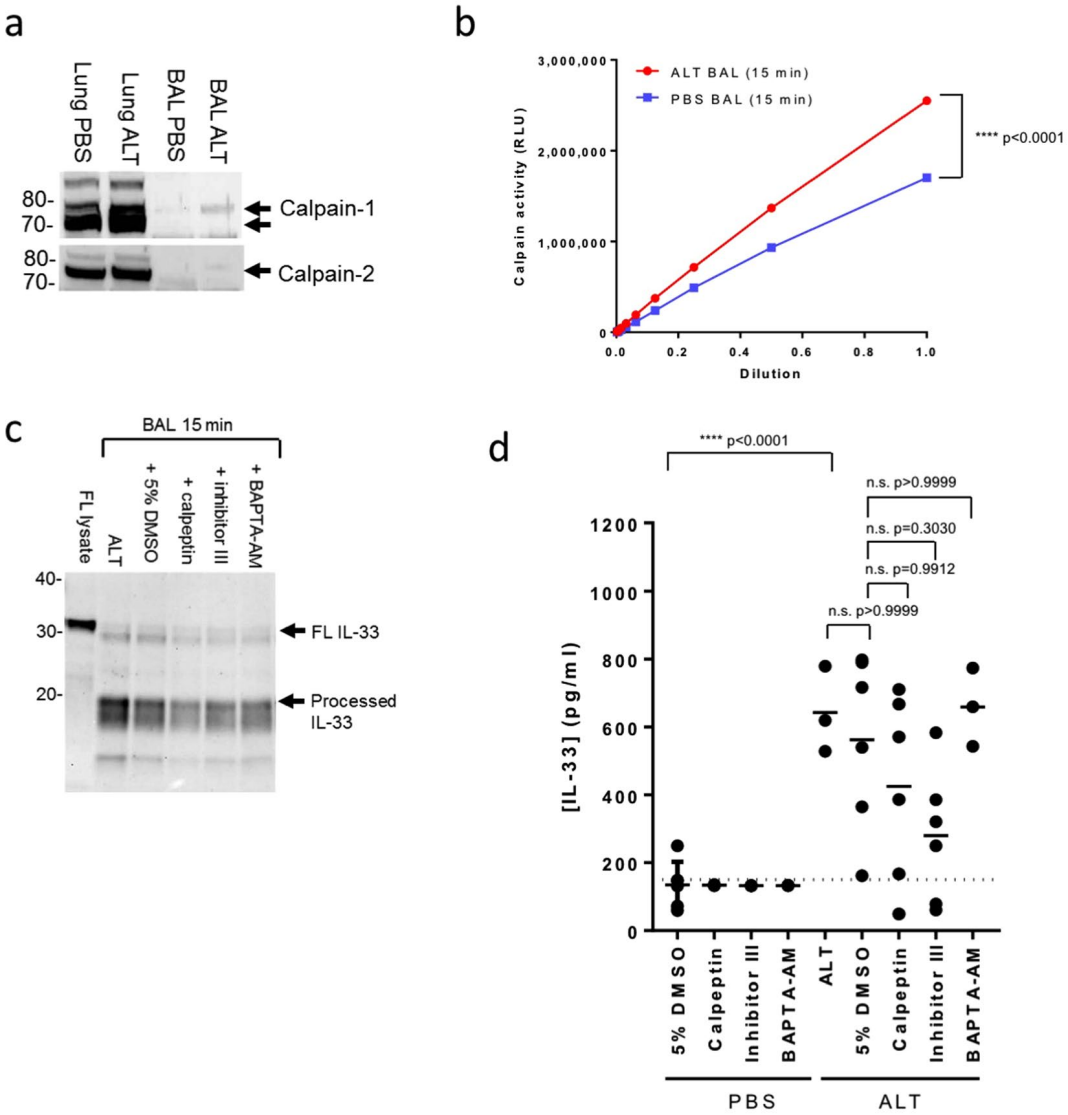
ALT-driven IL-33 processing *in vivo* is not dependent on calpain proteases. (**a**) Western blot of calpain-1 (upper panel) and -2 (lower panel) in mouse lung homogenates and BAL (pooled n = 3–4 mice/group) 30 min after ALT or PBS challenge. (**b**) Protease activity, measured using a calpain peptide substrate, in BAL (pooled n = 3–4 mice/group) collected 15 min after ALT or PBS challenge. RLU, relative light units. Data points are mean ± SEM. Statistical analysis: two-way ANOVA test, Tukey’s post-test, F = 1464, degrees of freedom = 10. ****P < 0.0001 for ALT v PBS group for undiluted samples. (**c**) Western blot of IL-33 in BAL (pooled n = 3–4 mice/group) 15 min after ALT challenge with and without co-administration of calpeptin, calpain inhibitor III, BAPTA-AM or 5% DMSO. Controls: FL lysate, lysate of CHO cells transfected with full length mouse IL-33. (**d**) Concentration of IL-33 (pg/ml) in BAL 15 min after ALT or PBS challenge with and without co-administration of calpeptin, calpain inhibitor III, BAPTA-AM or 5% DMSO. Controls: FL lysate, lysate of CHO cells transfected with full length mouse IL-33. Lower limit of detection is indicated by dotted line. Data points are mean ± SEM. Statistical analysis: one way ANOVA test, Tukey’s post-test, F = 6.182, degrees of freedom = 9. n.s.: non-significant. Data is pooled from n = 3 independent studies (**d**). Representative of n = 3 independent experiments (**a–c**). Western blot images (**a** and **c**) have been cropped for clarity with full blots presented in Figs S10 and S11.

We investigated whether calpains process IL-33 during ALT-driven epithelial damage *in vivo*. Despite evidence of increased calpain release (Figs 4a, S10) and activity (Fig. 4b) in BAL from ALT compared to PBS treated mice, we were unable to inhibit IL-33 processing *in vivo* with calpeptin, inhibitor III and BAPTA-AM (Figs 4c, S11). Inhibitors alone did not cause IL-33 release (Fig. 4d).

Collectively these data show that calpains can process IL-33 to mature forms with increased alarmin activity. Although calpains are co-released with IL-33 during epithelial damage, they do not appear to have a dominant role in IL-33 processing following ALT challenge *in vivo*.

### ALT-derived cysteine protease activities can directly process IL-33 to enhance its alarmin activity

**Figure 5:**
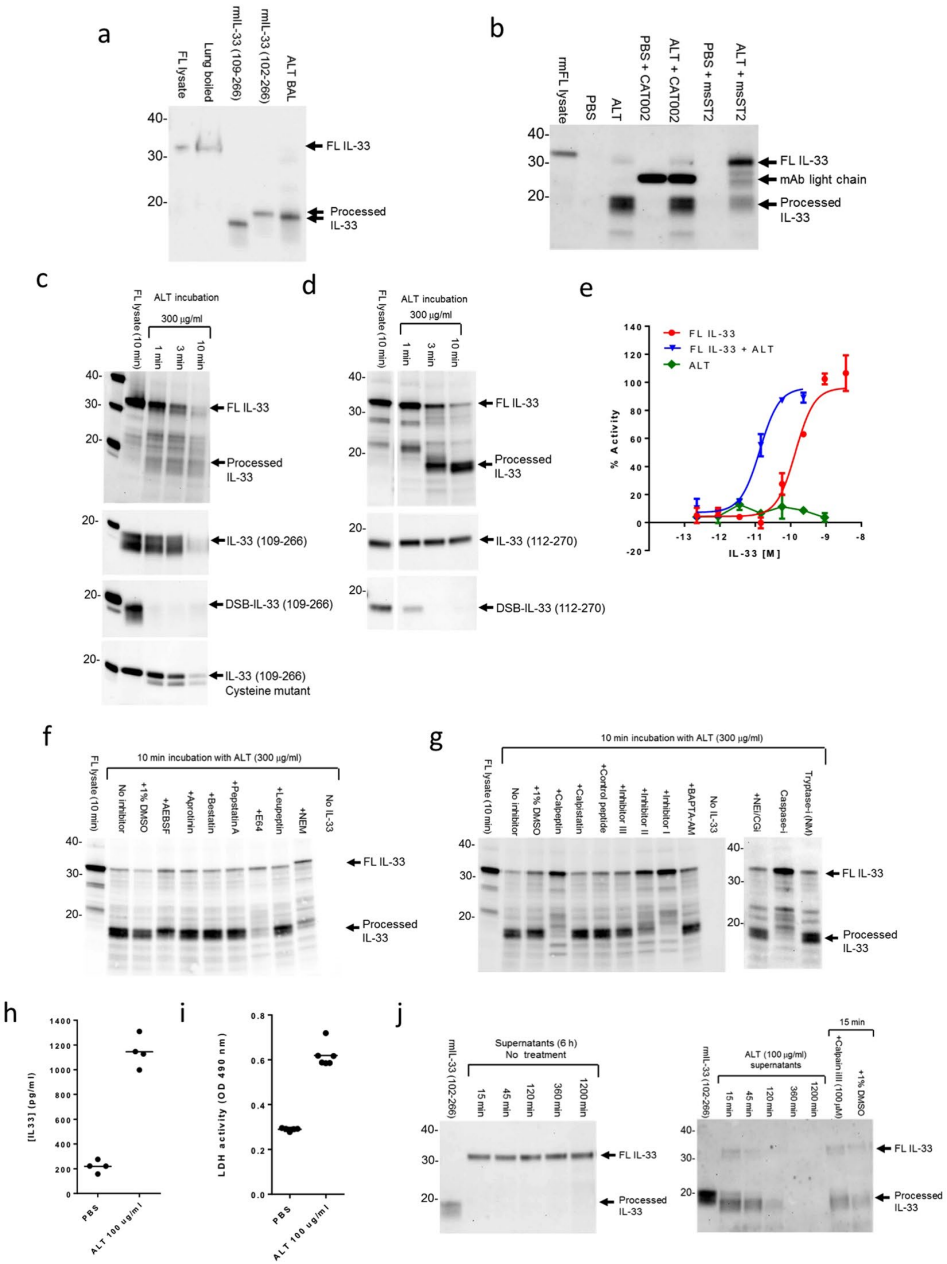
ALT-derived protease activities process IL-33 and increase its activity. (**a**) Western blot of IL-33 in mouse lung (boiled in SDS-PAGE buffer) and BAL 15 min after ALT challenge. Controls: FL IL-33, lysate of CHO cells transfected with full length mouse IL-33; rmIL-33 (102–266 and 109–266), recombinant mouse IL-33 (102- and 109-266 aa). (**b**) Western blot of IL-33 in BAL 15 min after ALT or PBS challenge. Mice were dosed i.p with recombinant mouse sST2-Fc or CAT-002 (control) for 30 min prior to i.n. ALT or PBS challenge and BAL collected after 15 min. Controls as (a). (**c**) Western blot of rmFL IL-33 lysate, rmIL-33 (109-266 aa), DSB-rmIL-33 (109-266 aa) and cysteine mutant rmIL-33 (109-266 aa) after incubation with 300 μg/ml ALT. (**d**) Western blot of rhFL IL-33 lysate, rhIL-33 (112-270 aa), DSB-rmIL-33 (112-270 aa) before and after incubations with 300 μg/ml ALT for 1-10 min. (**e**) Relative NFkB p65/RelA translocation in HUVECs 30 min after stimulation with rhFL IL-33 lysate and FL IL-33 pre-incubated with ALT. Data points are mean ± SEM of duplicate determinations. %Activity is calculated relative to signal of 3 ng/ml rhIL-33 (112-270 aa). (**f**) Western blot of rhFL IL-33 lysate before and after incubation with 300 μg/ml ALT for 10 min. rhFL IL-33 was pre-incubated with protease inhibitors or BAPTA-AM, for 15 min prior to addition of ALT. Details of protease inhibitors are described in legend for Fig. 3c. (**g**) Western blot of rhFL IL-33 lysate with and without incubation with 300 μg/ml ALT or PBS for 10 min. Controls: FL lysate, lysate of HEK cells transfected with full length human IL-33. Details of protease inhibitors are described in legend for Fig. 3d. (**h**) Concentration of IL-33 (pg/ml) in mouse CMT-64 cell supernatants 30 min after 100 μg/ml ALT or PBS challenge. (**i**) Lactate dehydrogenase (LDH) activity in mouse CMT-64 cell supernatants 6 h after 100 μg/ml ALT or PBS challenge. (**j**) Western blot of mouse CMT-64 cell supernatants 15-1200 min with (right panel) or without (left panel) 100 μg/ml ALT. ALT treatment was performed with or without pre-incubation for 15 min with 100 μg/ml calpain inhibitor III or 1% DMSO. Controls as (**a**). Representative of n = 3 (**a–e**) and n = 2 independent experiments (**f–j**). Western blot images (**a**–**d,f,g,j**) have been cropped for clarity with full blots presented in Figs S12–16.

Mature forms of IL-33 in the BAL and lung of ALT challenged mice appear to migrate slightly faster than recombinant mIL-33 (102–266 aa), potentially indicating processing at an alternative site to that employed by endogenous proteases (Figs 5a, S1b and S12). In addition, we discovered rather surprisingly that recombinant mouse sST2 inhibited generation of ~18 kDa IL-33 after ALT challenge in mice (Fig. 5b, S12), suggestive that exogenous proteins could influence IL-33 processing. Putting these data together with our earlier findings that i.n. dosing of ALT caused damage to the lung epithelium, we hypothesised that extracellular proteins might process FL IL-33 from ALT-damaged airway epithelial cells during its release.

Protease activities present in ALT extract can degrade epithelial barriers, including tight junction proteins, and activate epithelial cells^22,28,29^ thus we explored whether ALT proteases themselves might directly process IL-33 associated with damaged epithelial cells and during its release. Using a concentration of ALT similar to that used *in vivo* (300 μg/ml), ALT extract directly processed FL rmIL-33 to an ~18 kDa form (Figs 5c, S13). Mature mouse IL-33 was susceptible to further degradation by ALT (Figs 5c, S13). Oxidation of mature mouse IL-33 increased the susceptibility to degradation whereas oxidation-resistant mature mouse IL-33 was degraded at a similar rate to wild type mature mouse IL-33 (Figs 5c, S13). Human FL IL-33 was also rapidly processed to ~18–19 kDa forms on incubation with ALT (300 μg/ml) (Figs 5d, S14), which increased its activity by up to 10-fold (Fig. 5e). In contrast to mice, degradation of mature human IL-33 was dependent on its oxidation (Fig. 5d). Unlike FL IL-33, ALT treatment of the reduced, mature human IL-33 (112–270 aa) did not impact its activity (Fig. S4a).

ALT serine protease activities are known to increase IL-33 release^22^ but, surprisingly, serine protease inhibition (including aprotinin, AEBSF) had no effect on the ability of ALT to process FL IL-33 (Figs 5f, S15). In contrast, cysteine protease inhibitors (E64 and NEM) substantially inhibited IL-33 processing. Of more selective inhibitors, calpeptin, calpain inhibitors I and II, and a pan-caspase inhibitor (caspase-i), but not calpain inhibitor III, BAPTA-AM, tryptase, NE and CG inhibitors inhibited IL-33 processing (Figs 5g, S15).

These data are consistent with cysteine protease activities in ALT extract being capable of processing recombinant IL-33 *in vitro*. However, the nature of the ALT-derived cysteine protease activities appears distinct from calcium-dependent calpain proteases.

### ALT extract protease activities process endogenous IL-33 and increase its release

We investigated whether ALT would also process endogenous IL-33. *In vitro*, ALT (100 μg/ml) caused increased release of IL-33 and LDH from CMT-64 cells (Fig. 5h,i) as well as rapid processing of IL-33 to a mature ~18 kDa form, and subsequent degradation of IL-33 (Fig. 5j). ALT also caused the processing and release of IL-33 from NHBE cells (Fig. S4b,c). Unlike calpain regulated processing observed in detergent or ionomycin-damaged epithelial cells (Figs 3d, S3d), ALT-mediated IL-33 cleavage was not inhibited by calpain inhibitor III (Figs 5j, S16). *In vivo*, we were unable to impact ALT-driven IL-33 processing, even with broad-spectrum protease inhibitors (Fig. S4d,e). However, a serine protease inhibitor (AEBSF) reduced IL-33 release into BAL (Fig. S4d,e) in a predominantly proteinase-activated receptor-2 (PAR-2) independent manner (Fig. S4f,g).

Taken together our data indicate that ALT extract proteases can induce the release and processing of IL-33. ALT extract serine proteases drive IL-33 release from airway epithelial cells, whereas cysteine proteases access damaged epithelial cells and proteolytically activate IL-33. Lastly ALT-derived proteases can degrade oxidized mature forms of IL-33.

### FL and N-terminally processed forms of IL-33 are detected in human lung tissue

**Figure 6:**
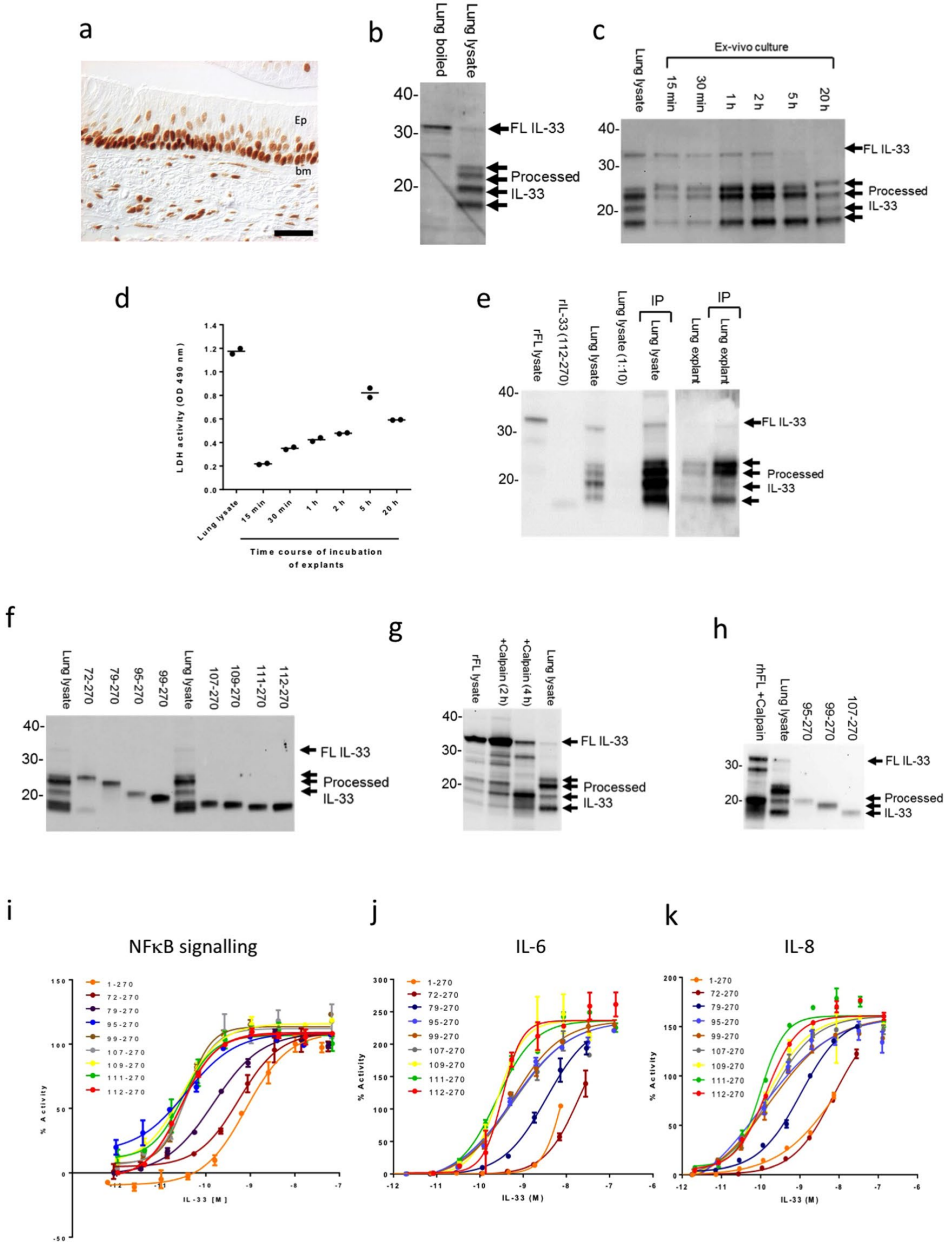
FL and N-terminal processed forms of IL-33 are detected in the human lung. (**a**) Bright field micrograph demonstrating IL-33 immunoreactivity (brown DAB chromogen) in small airways of lungs from severe COPD patient (GOLD stage 4). Ep: epithelium; bm basement membrane. Scale bar = 40 μm. (**b**) Western blot of IL-33 expression in human COPD lung tissue (boiled in SDS-PAGE buffer) and lung tissue lysate. (**c**) Western blot of IL-33 expression in human COPD lung tissue and lung explant supernatants (15 min to 20 h after incubation). (**d**) Lactate dehydrogenase (LDH) activity in human lung explant supernatants (samples as (c). (**e**) Immunoprecipitation (IP) and western blot of IL-33 in human COPD lung lysate and explant supernatants (2 h). IL-33 was IP using anti-IL-33 mAb (#640050) and western blot performed with anti-IL-33 Ab (AF3625). Controls: rFL lysate, lysate of HEK cells transfected with full length human IL-33. (**f**) Western blot of mature forms rhIL-33 (72-, 79-, 95-, 99-, 107-, 109-, 111-, 112-270 aa) and human lung lysate. (**g**) Western blot of rhFL IL-33 lysate, with or without incubation for 2-4 h with calpain, and human lung lysate. Controls as (e). (**h**) Western blot of rhFL IL-33 lysate, incubated for 2 h with calpain, lung lysate and mature forms of rhIL-33 (95-, 99-, 107-270 aa). (**i**) Relative NFkB p65/RelA translocation in HUVECs 30 min after stimulation with rhIL-33 (1-, 72-, 79-, 95-, 99-, 107-, 109-, 111-, 112-270 aa). Data points are mean ± SEM of duplicate determinations. % Activity is calculated relative to signal of 3 ng/ml rhIL-33 (112-270 aa). Relative IL-6 (**j**) and IL-8 (**k**) release from HUVECs 20 h after stimulation with rhIL-33 (1-, 72-, 79-, 95-, 99-, 107-, 109-, 111-, 112-270 aa). Data points as (j). Data points are mean ± SEM of duplicate determinations. % Activity is calculated relative to signal of 30 ng/ml rhIL-33 (112-270 aa). Representative of n = 2 (**b,d,f–j**), n = 3 (**a,k**) and n = 4 independent experiments (**c**). Western blot images (**b,c,e–h**) have been cropped for clarity with full blots presented in Figs S17–S20.

The nature of endogenous IL-33 released from human tissue has remained poorly characterized. Various different IL-33 processing sites have been proposed^14,15^ and we sought to determine if corresponding mature forms of IL-33 could be detected. Human lung tissue contains high levels of IL-33^17^ predominantly localised to the nuclei of cells in airway epithelium with lower levels in structural cells of subepithelial tissue (Fig. 6a). Consistent with this staining pattern, IL-33 was in predominantly a FL form (~31 kDa) in human lung tissue (Fig. 6b) but detected in FL and at least four mature forms with MWs of ~18, 20, 22 and 23 kDa in damaged lung following homogenization (Fig. 6b) or explant culture (Fig. 6c,d). No obvious difference in the pattern of IL-33 forms was detected across COPD (Figs 6b, S17), asthmatic, or smoking status (Fig. S5a). IL-33 in lung tissue lysate and in explant supernatants immunoprecipitated with IL-33 mAb #640050 (Figs 6e, S5b and S18) indicating mature forms of endogenous human IL-33 were N-terminal processed.

To investigate more precisely the observed human IL-33 species, recombinant mature forms of IL-33 were expressed according to proposed IL-33 cleavage sites^14,15^ (Supplementary Table 1) and compared with those released from damaged lung. The apparent MW of endogenous forms of IL-33 were consistent with processing after residue 71, 78, 94, 106, 108 and 110, but not 98 (Figs 6f, S19). Processing by calpain (Figs 6g,h, S19–20) or CG (Fig. S5c) generated an IL-33 form that aligned with IL-33 (95–270 aa) present in human lung lysate. However, no form corresponding to the NE-generated IL-33 (99–270 aa) was observed in either human lung lysate or *ex vivo* culture (Fig. S5d).

### Mature forms of IL-33 are up to ~60-fold more active than FL IL-33

We comprehensively compared the potency of purified human FL IL-33 (1–270 aa) with N-terminal truncated forms. Mature forms of IL-33 with an N-terminus at or after residue 95 showed similar activity to each other (~20–60 fold more potent than FL IL-33 across several different functional assays) (Fig. 6i–k). In contrast IL-33 (72–270 aa) had similar activity to FL IL-33 and IL-33 (79–270 aa) was only 3–8-fold more potent.

In summary, we have demonstrated that endogenous human IL-33 is N-terminally processed at multiple sites leading to generation of a range of forms with differing biological activity. Mature IL-33 forms generated in human lung tissue are compatible with some, but not all, previously proposed proteolytic mechanisms.

## Discussion

IL-33 is an IL-1 cytokine family alarmin that is strongly linked by genetic and functional studies to epithelial and endothelial barrier injury, allergic and inflammatory diseases^2,3^. Surprisingly, given the strong interest in IL-33 function and efforts to target it therapeutically, the mechanisms regulating IL-33 activity have remained controversial in comparison to other IL-1 family cytokines^13,30^. While FL IL-33 has some biological activity, IL-33 can be proteolytically processed into shorter N-terminal truncated forms^13,14^ although the role of these different forms and mechanisms for their generation *in vivo* are uncertain^2^.

Here we sought to clarify whether selected immune cell proteases implicated in IL-33 processing *in vitro*^14,15^ process endogenous IL-33 during airway inflammation *in vivo* and, in doing so, identified alternative pathways for IL-33 processing and activation. We report for the first time that IL-33 activity can be enhanced by proteolytic mechanisms involving exogenous allergen proteases as well as endogenous epithelial cell-derived calpains. We suggest that IL-33 can recognise a variety of allergen-, pathogen-, necrosis- and inflammation-associated proteolytic activities that can modify IL-33 activity to initiate and fine -tune immune responses.

Sensitivity to fungal allergens is a prominent cause of allergic asthma with incidence of severe asthma associated with fungal sensitisation estimated to be as high as 30%^31^. The fungal allergens in *Alternaria alternata* (ALT) are particularly linked with asthma severity, hospitalization and exacerbations^32–35^. Lung ALT challenge in mice initiates release and processing of IL-33 and IL-33 dependent allergic airway inflammatory responses^9,17,22^. To understand the contribution of neutrophils, mast cells and other immune cell proteases in the processing of IL-33 released after ALT challenge *in vivo* we utilised mice deficient in DPP-1, which lack NE, CG, PR-3, gzmB, and chymase activities as well as a ~75% reduction in tryptase activity^19–21^. In addition, we performed studies in mice deficient in mast cell chymase and tryptase activities^23^. Here we discovered, in contrast to previous reports, that IL-33 processing can occur independent of these immune cell proteases. However, we cannot completely rule out that immune cell proteases, activated independently of DPP-1 and mast cells, potentially might contribute to IL-33 processing in ALT challenged mice. *Cstc*^−/−^ mice have some gzmB activity that is dependent on cathepsin H activity^36^. However neutrophil serine proteases are completely dependent on DPP-1 for activation in mice^19^ and humans^37^.

In *Ctsc*^−/−^ mice IL-33 processing did not occur during the peak of ALT-driven immune cell infiltration into the lung but instead correlated with ALT-driven damage to the airway epithelium. We discovered that IL-33 released from damaged airway epithelial cells was processed by calpains, necrosis-activated Ca^2+^-dependent proteases^38–40^, that were previously shown to cleave recombinant IL-33 in a transfected cell system^18^. In addition, we have demonstrated for the first time that processing of IL-33 by calpain-1 occurred within its activation domain^14^ to enhance its biological potency. We propose that human IL-33 is likely processed by calpain between Phe-94 and Ala-95, at the same site proposed for CG but distinct from that proposed for NE^15^. The proposed calpain processing site is consistent with the preference for large hydrophobic amino acids at the P1 position of calpain cleavage sites that are present in human IL-1α (Phe-118 and Ser-119)^41^ and optimized peptide substrates for calpains (e.g. Glu-Pro-Leu-Phe/Ala-Glu-Arg-Lys)^42^. Based on this premise and the apparent MW of processed IL-33 from mouse epithelial cells we speculate calpains might process mouse IL-33 between Phe-101 and Ala-102 at the same site as NE and CG^13^.

We investigated if calpains were responsible for IL-33 processing *in vivo* in ALT-challenged mice. Calpain-1 and -2 were co-released with IL-33 into BAL consistent with previous work showing release from damaged tissues^43^. However, at doses previously shown to inhibit endogenous calpain activities in mice^44,45^, the cell permeable calpain inhibitors, calpain inhibitor III and calpeptin^25,46^ did not inhibit IL-33 processing in our ALT challenge model *in vivo*. Unfortunately, due to the complexity of calpains^47^ and essential roles in embryonic development^48,49^ we were unable to investigate the role of these proteases more conclusively using gene-targeting approaches. It is possible that the role of calpains in processing endogenous IL-1α activity *in vivo* has not been investigated further due to the same challenges and it is hoped that in future, conditional knockout mice might provide an avenue to explore this biology^50^.

In pursuit of alternative mechanisms of IL-33 processing we unexpectedly discovered that exogenous mouse sST2 inhibits the generation of mature endogenous IL-33 after ALT challenge in mice presumably by binding the IL-1 like cytokine domain of FL IL-33. Recently alarmin release inhibitor protein (HpARI)^51^, an exogenous protein from *H. polygyrus*, was shown to access necrotic epithelial cells, bind IL-33 and interfere with its release. In a similar way, it is conceivable that sST2 might access necrotic airway epithelial cells, bind FL IL-33, and inhibit both proteolytic activation and binding to the ST2 receptor. Although the structure of the N-terminal domain of FL IL-33 is unknown it is plausible that sST2 sterically hinders the access of proteases to the proteolytic activation domain of IL-33^14^.

We hypothesised that ALT extract proteases might enter damaged cells and target IL-33. We discovered that ALT extract could rapidly process FL IL-33 to mature forms in biochemical and epithelial cell based assays. Importantly ALT-driven processing of IL-33 increased the potency of IL-33 by ~10-fold. Unexpectedly, this processing appeared to be driven by cysteine protease activities in contrast to ALT-derived serine proteases that increased IL-33 release. Using broad spectrum serine protease inhibitors, we confirmed the roles of serine proteases on IL-33 release *in vivo*. However, we were unable to confirm a role for ALT-derived cysteine proteases on IL-33 processing *in vivo*. The reasons for this discrepancy are unclear however we speculate a very high % protease inhibition might be required to block processing whereas a modest reduction in serine protease activity might be sufficient to reduce membrane damage and permeability to enzymes. However, we cannot rule out a compensatory role for endogenous proteases or that the cysteine protease inhibitors have poor *in vivo* pharmacokinetic properties.

Mature forms of IL-33 can be rapidly inactivated in the extracellular environment by disulphide bonding (DSB) of critical cysteine residues^17^. Here we found that ALT-derived proteases, in addition to processing IL-33 to more potent forms, could degrade mature IL-33. Whilst ALT-driven degradation of mature human IL-33 was dependent on its oxidation, mature mouse IL-33 could be degraded in both reduced and oxidized forms though oxidation increased susceptibility to degradation. DSB-IL-33 might be particularly liable to proteolytic degradation as it adopts a relatively unstructured conformation, compared to reduced IL-33^17^. Indeed, degradation of DSB-IL-33 by ALT might contribute to the rapid decline in IL-33 levels in BAL 60 min after ALT challenge.

Future work will need to further investigate the potential interplay of processing and cysteine oxidation in regulating the activity of FL and mature IL-33. It has been suggested that immune cell proteases also have dual functions in proteolytic activation and degradation of cytokines including IL-33^52–54^. However, we did not see changes in IL-33 levels in unchallenged, ALT or PBS challenged lung tissues from DPP-1 and mast cell deficient mice.

ALT and other pathogenic fungal allergens that are associated with allergic airway disease express high levels of protease activities that appears to differentiate them from more benign allergens^22,29,55^. Interestingly only i.n. ALT challenge, but not other allergens, resulted in robust IL-33 release^22^. However, the biological mechanisms for how ALT induces IL-33 release are not well understood. ALT serine protease activity causes epithelial barrier disruption, epithelial cell detachment and production of IL-6 and IL-8^28,29^. We speculate that, secondary to IL-33 release, ALT protease driven IL-33 activation is a hitherto unsuspected mechanism that increases the impact of IL-33 on the innate immune system during our ALT-driven allergic inflammation model and could play a similar role during allergen invoked asthma exacerbations. However further work is required to understand if exposure to ALT spores, rather than ALT extracts, can drive IL-33 activation and release and contributes to the rapid decline in respiratory function and initiate rapid onset of severe disease^56^. It is reported that IL-1β can be proteolytically activated by fungal (*Candida albicans*) and bacterial (*Treponema denticola* and *Streptococcus pyogenes*) proteases to elicit inflammatory responses^57–59^. As most microorganisms secrete an array of proteases, it has been suggested that pathogen-evoked mechanisms for proteolytic activation of IL-1 family cytokines might be active during allergen challenges or infection^60^. We speculate that proteolytic activation of IL-33 and other IL-1 family cytokines by additional allergen or pathogen-derived proteases remain to be elucidated.

We reliably found ALT (4 different batches) could induce epithelial cell cytotoxicity *in vitro* and *in vivo*, as measured by LDH release. We found cell damage was necessary for release of IL-33 in contrast to a previous study^9^ using similar concentrations of ALT. In line with our previous report^17^ we also observed ALT-driven IL-33 processing *in vitro* and *in vivo*. We suggest inconsistencies in reported activities of ALT could be due to batch to batch variance in extract composition, supplier and storage conditions.

Proteases present during tissue damage can signal to cells via proteinase-activated receptors (PAR) to mediate inflammation^61^. Studies with PAR-2 antagonists have suggested ALT-derived serine proteases drive lung inflammation, airway epithelial cell activation^62,63^, and IL-33 release^22^ in a PAR-2 dependent manner. However, in contrast, we found that release and processing of IL-33 in ALT challenged *Par2*^−/−^ mice^64^ appeared to be similar to WT mice. The reasons for these conflicting results are unclear, but could be due to non-selective activities of PAR-2 antagonists or variations in ALT batches as discussed above. Consistent with our results in *Par2*^−/−^ mice we found ALT-driven calcium signalling in mouse and human airway epithelial cells *in vitro* was not blocked by PAR-2 neutralising mAb or peptide antagonists, and ALT-driven calcium signalling was similar in PAR-2 overexpressing and PAR-2 deficient cells (unpublished observations).

In these studies, we have uniquely characterised the endogenous forms of IL-33 in human lung tissue and correlated with proposed processing mechanisms^14,15^. IL-33 released from human lung tissue was detectable in FL and at least 4 N-terminally processed forms consistent with proposed processing sites for calpain, CG, tryptase, chymase, gzmB and ALT proteases. Somewhat unexpectedly we did not detect forms of IL-33 corresponding to processing sites proposed for NE^15^ or potential caspase-cleaved forms of IL-33^8,10^. We cannot rule out that proteases can sequentially process IL-33, although the most N-terminal processed forms of endogenous IL-33, potentially corresponding to 72- and 79–270 aa forms, are stable enough to be readily detectable in human lung samples. However, it is also possible that partially truncated forms of IL-33 make poor substrates for further proteolytic processing.

A limitation of this study is that we have induced processing in *ex-vivo* human lung by mechanical damage (homogenization or explant cutting). Thus, we cannot rule out that different mature IL-33 forms might be detected in biological fluids such as sputum or serum. Nevertheless, overall our data strongly suggests that IL-33 can be released as a FL protein and in multiple processed forms. Interestingly, release of FL IL-33 suggests that removal of the N-terminal domain is not an absolute requirement for IL-33 release despite the importance of the N-terminal domain in tethering IL-33 to chromatin within cell nuclei^65^ and regulating its release^12^. The relative contribution of different mechanisms for the proteolytic activation of IL-33 *in vivo* is likely to depend on the biological or pathological context. For example, proteolytic activation of IL-1β *in vivo* is more dependent on CG, NE and PR-3 than caspase-1 in a neutrophil-dominated model of arthritis^66,67^. As the IL-33 activation domain (66–111 aa)^14^ appears highly susceptible to proteolysis^14^ it is probable that additional proteases can generate mature forms of IL-33.

There is a paucity of studies on the proteolytically processed forms of IL-33 detected in human biological fluids possibly due to the low levels of IL-33 (pg/ml). Pastorelli *et al*. detected 20–22 kDa processed forms of IL-33 in serum from inflammatory bowel disease patients and healthy individuals^68^ that are consistent with the size of mature forms of IL-33 we detected in the human lung. Zhang *et al*. appeared to detect FL and processed forms of IL-33 in human serum although the MW of these IL-33 forms were not clear^69^. High affinity antibodies and high-resolution techniques such as mass spectroscopy would be of great interest to characterise native forms of human IL-33. Although alternative splicing has been suggested to generate different MW forms of IL-33^70^ we found IL-33 in unchallenged lung tissue was primarily present as a FL protein.

Finally, we have advanced the understanding of the functional importance of IL-33 processing on increasing IL-33 biological potency. To ensure native protein folding we expressed FL IL-33 in mammalian cells, rather in a cell free *in vitro* translation system^14,15^, and showed, for the first time, that calpain and ALT extract proteases can increase the potency of IL-33 similarly to immune cell proteases. We comprehensively compared the potencies of highly purified FL and mature forms of IL-33 and demonstrated, irrespective of the protease involved, mature forms of IL-33 with an N-terminus at or after residue 95 were 26–50-fold more potent than FL IL-33. In contrast, an N-terminus at residue 72 had similar activity to FL IL-33, while N-terminal of residue 79 enhanced activity only 3–8-fold. The increased potency of shorter forms of IL-33 suggests the N-terminal domain might partly obstruct IL-33 binding to ST2 and removal of part or all of this domain can permit a more stable interaction. It has been suggested that the highly basic N-terminal domain of IL-33 might interfere with the binding of the acidic cytokine domain of IL-33 to ST2^14^. It is plausible that IL-33 processing alters the conformation of IL-33 to increase affinity for ST2 in a similar way to IL-1α^71^. However, the structure of FL IL-33 and measurement of IL-33-ST2 affinities are required to confirm these mechanisms.

**Figure 7:**
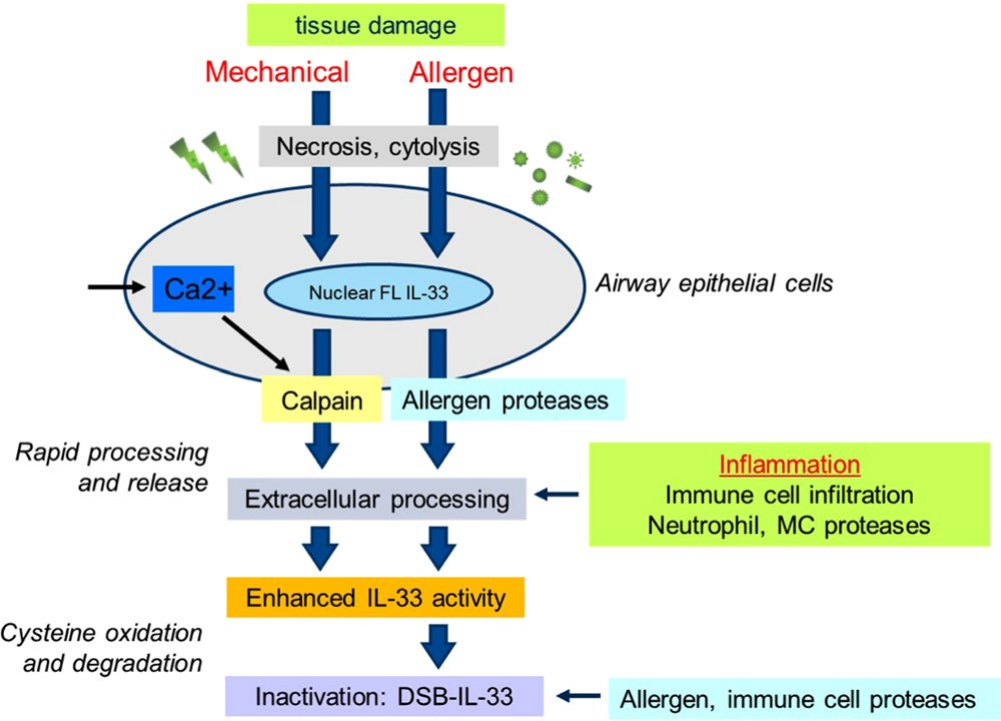
Hypothesis for the proteolytic regulation of IL-33 activity during injury to airway epithelium. Full length IL-33 is stored in the nucleus of airway epithelial cells. Mechanical or allergen induced cell damage induces epithelial cell necrosis, loss of cell membrane integrity and cytolysis. Increased intracellular levels of Ca^2+^ ions at sites of epithelial damage trigger calpain protease activity. Calpains proteolytically activate IL-33 during its release from damaged epithelial cells. Airborne allergen-derived proteases can damage epithelial cells, enhance IL-33 release and proteolytically activate IL-33. In addition, during inflammation proteases from infiltrating immune cells can also contribute to the extracellular proteolytic activation of IL-33. Subsequently mature forms of IL-33 are rapidly inactivated by cysteine oxidation and degraded by allergen or possibly immune cell proteases. Thus IL-33 can utilise different proteolytic activities present during airway epithelial damage to be rapidly activated and inactivated and limit the duration of its alarmin activity.

In summary, we have identified hitherto unknown mechanisms by which IL-33 can be activated during epithelial injury to regulate the magnitude and duration of immune responses. Contrary to current thinking we found proteolytic activation of IL-33 can occur independent of immune cell proteases *in vivo*. Of particular interest, we show for the first time that fungal allergen proteases can directly amplify functional activity of IL-33. We propose an updated hypothesis for the regulation of IL-33 activity by proteolytic processing (Fig. 7). Mechanical damage to the epithelial barrier increases intracellular Ca^2+^ levels^72^ that trigger necrosis-associated calpain proteases to proteolytically enhance IL-33 activity during its release. Similarly, pathogenic allergen-derived proteases that access damaged epithelial cells can proteolytically enhance IL-33 activity. In addition, during the inflammatory response to injury proteases from infiltrating immune cells can process released FL IL-33 in the extracellular space to further amplify levels of IL-33 activity. Finally, allergen, and possibly immune cell protease activities, can degrade mature oxidized forms of released IL-33. In conclusion, we propose IL-33 activity is regulated by the proteolytic as well as the oxidative milieu during tissue damage to rapidly escalate and diminish its alarmin activity to fine tune the magnitude and duration of immune responses.

## Methods

### Reagents

4-(2-Aminoethyl) benzenesulfonyl fluoride hydrochlorine (AEBSF, A8456), aprotinin (A1153), bestatin (Sigma B8385), pepstatin A (P5318), N-(trans-Epoxysuccinyl)-L-leucine 4-guanidinobutylamide (E64, E3132), leupeptin (L2884), N-ethylmaleimide (NEM, E3876), calpeptin (C8999), calpain inhibitor-I (A6185), -II (A6060), -III (M6690), pan-caspase inhibitor (V116), BAPTA-AM (A1076), ionomycin (I0634) were supplied by Sigma. Calpistatin peptide (208902), negative control peptide (208904), elastase inhibitor IV (324759) and cathepsin G inhibitor I (219372) were supplied by Calbiochem. Tryptase inhibitor nafamostat mesylate (NM, 3081) was supplied by Tocris. AEBSF, aprotinin, bestatin, pepstatin A, E64, leupeptin and NEM were used *in vitro* at concentrations according to manufacturer’s instructions (Sigma Inhib1). Calpeptin, calpain, NE, CG, caspase and tryptase inhibitors, BAPTA-AM were used at 100 μM during *in vitro* studies. Calpistatin and control peptide were used at 10 μM during *in vitro* studies. Doses of inhibitors used *in vivo* and details of all other reagents used are described in other method sections.

### Recombinant proteins

Recombinant IL-33 proteins were generated according to human IL-33 (accession number Swiss-Prot O95760) or mouse IL-33 (accession number Swiss-Prot Q8BVZ5) consensus sequences. Human IL-33 (1-, 72-, 79-, 95-, 99-, 107-, 109-, 111-, 112–270 aa) and mouse IL-33 (102-, 109–266 aa and a mutant 109–266 aa form in which all 4 cysteines were replaced by serines) were modified to contain sequences for an N-terminal GST-His-SUMO, MBP-His-SUMO or FLAG^®^ 10xHis tag (DYKDDDDKAAHHHHHHHHHH). Proteins were expressed in *E.coli* and purified in presence of 2 mM DTT. For untagged proteins, the GST or MBP fusion proteins were cleaved using SENP1, re-purified via Nickel affinity chromatography and size exclusion chromatography (SEC), and eluted into PBS. DSB-IL-33 and IL-33 cysteine to serine mutant proteins were generated as previously described^17^. HEK-EBNA cells were transiently transfected with human IL-33 (1–270 aa) or water (mock), cell lysates cleared and harvested (10e8 cells/ml) into PBS. CHO-CEP6 cells were transiently transfected with mouse IL-33 (1–266 aa) and cell lysates cleared and harvested (10e8/ml) into PBS. Human IgG_1_ antibodies (H338L293, CAT-002) were generated in CHO cells. ST2-Fc fusion proteins were purchased from R&D systems (523-ST and 1004-MR).

### IL-33 detection reagents

IL-33 antibodies were isolated by phage display (#H338L293, #640050, #640104-3) or purchased from commercial suppliers (R&D Systems AF3625, AF3626). IL-33 was quantified by Duoset ELISA (R&D Systems DY3625, DY3626), according to manufacturer’s instructions, or in house ELISAs where #640050 (2 μg/mL) was used as capture antibody and detected with biotinylated goat polyclonal (R&D Systems BAF3625, 500 ng/mL).

### Mice

Wild-type BALB/c and C57BL/6 J mice were purchased from Charles River, UK. Generation of BALB/c *Il33*^−/−^ mice has been described previously^73^. C57BL/6 J *Ctsc*^−/−^ mice were generated by Deltagen Inc. and supplied by AstraZeneca. *Ctsc*^−/−^ mice gene targeting strategy, genotyping and basic phenotyping are described at http://www.informatics.jax.org/allele/MGI:3604533. C57BL/6 J *Par2*^−/−^ mice^64^ were purchased from Jackson Labs. BALB/c Cre-Master mice^23^ were licenced from Prof H. R. Rodewald, German Cancer Research Centre, Heidelberg. For experiments with *Ctsc*^−/−^ and Cre-Master mice wild type littermates were used as wild type controls. Mice were bred and maintained under specific pathogen-free conditions in accredited animal facilities at MRC-Harwell, UK.

As required by the Animals (Scientific Procedures) Act 1986, all in *vivo* work was carried out in accordance with guidelines and regulations of a UK Home Office Project Licence, which had been reviewed and approved by the Babraham Institute Animal Welfare and Ethical Review Body (AWERB). Male and female mice (6-10 weeks old) were anaesthetized briefly with isofluorane and challenged with 25 µg of *Alternaria alternata* (ALT) extract (XPM1D3A25, Greer, Lenoir, NC), or PBS intranasally (i.n.) in a total volume of 50 µl. In some experiments mouse sST2-Fc (10 mg/kg) or CAT-002 mAb (10 mg/kg) were mixed with 25 µg ALT or PBS for 15 min before i.n. dosing. The broad-spectrum protease inhibitors AEBSF (2.5 mg/kg and 250 kg/kg), aprotinin (16 mg/kg), NEM (2.5 mg/kg), E64 (7.2 mg/kg) and leupeptin (10 mg/kg) were each pre-incubated with 25 µg ALT or PBS for 15 min before i.n. dosing. The calpain inhibitors calpeptin and calpain inhibitor III were dosed (5 mg/kg) intraperitoneally 30 min prior to an i.n. challenge with 25 µg ALT or PBS that had been mixed with calpeptin (5 mg/kg) or calpain inhibitor III (5 mg/kg). BAPTA-AM (5 mg/kg) was mixed with 25 µg ALT or PBS for 15 min before i.n. dosing. At selected time points (5 min to 24 h) after challenge, mice were terminally anaesthetised with pentobarbital sodium. Bronchoalveolar lavage fluid (BAL) was collected by lavage with PBS (0.3 ml, 0.3 ml & 0.4 ml) via tracheal cannula. BAL was centrifuged, cells counted and cell free supernatant was analysed for different cytokines (detailed in other methods sections), protease and lactate dehydrogenase (LDH) activities. Mouse lung tissue was boiled in SDS-PAGE buffer containing 2% beta-mercaptoethanol (100 mg tissue/ml) for 5 min and supernatants cleared by centrifugation (to remove any debris). Lung tissue lysates were prepared by homogenization of lung tissue (100 mg tissue/ml) in PBS on ice and supernatants cleared by centrifugation. IL-5 levels in BAL were detected by ELISA (Meso Scale Discovery, Rockville, MD). Differential cell counts (200 cells/slide) were performed on cytospin preparations stained with Diff-Quik (Fisher Scientific,UK). In the data presented the group sizes were set in order to generate sufficient material for *ex vivo* analyses and determine profiles of IL-33 forms. For inflammation readouts, sample size analysis was performed by using previous experimental data. Randomization was performed at the time of animal delivery and although formal blinding was not used, the dosing and sample collection was performed by technical staff not involved in protein generation or analysis.

### Human tissue samples

The study was approved by the NRES East of England (Cambridge East) Research Ethics Committee (reference number 08/H0304/56 + 5) and all methods were carried out in accordance with their guidelines and regulations. Tissue was donated with the informed consent of patients. Non-cancerous adjacent tissue from lung cancer patients and from lung transplant surgeries were supplied in Aqix RS-I medium (Aqix Ltd) on ice by Papworth Hospital NHS Trust Research Tissue Bank. The tissues used in this study were COPD, asthmatic, smoker, ex-and smoker patients. ~3 g of lung tissue was washed 3 × in PBS in a sterile Petradish (Costar) and ~0.5 cm^3^ tissue explants prepared using a sterilised metal cork borer. Lung tissue was boiled in SDS-PAGE buffer containing 2% beta-mercaptoethanol (400 mg tissue/ml) for 5 min and supernatants cleared by centrifugation (to remove any debris). Lung tissue lysates were prepared by homogenization of lung tissue (400 mg tissue/ml) in PBS on ice and supernatants cleared by centrifugation. Explants were incubated in PBS/0.1% BSA in wells of a 96-well tissue culture plate (Costar) for 15 min, 30 min, 1 h, 2 h, 5 h, and 20 h under standard tissue culture conditions. Each time point was performed with n = 8 replicates and pooled supernatant samples from each time point were cleared by centrifugation, aliquoted and snap frozen on dry ice. IL-33 forms in boiled lung tissue, lung lysates and explant supernatants were analysed by ELISA and western blot as described.

### Protease activity assays

Mouse bone marrow lysates (4e7 cells/ml) were prepared by perfusion of hind leg femurs with RPMI medium. Cells were washed in PBS (containing Ca2 + and Mg2 + ) and collected in 20 mM sodium acetate pH 5.5, 1 mM EDTA, 1% Triton X100. DPP-1 activity in mouse bone marrow lysates was determined by measuring the release of amino-methyl-coumarin (AMC) from the DPP-1 peptide substrate H-Gly-Arg-AMC (Bachem I1215) in 96-well assay plates (Nunc 475515) containing 50 μl cell lysate and 50 μl of 200 μM substrate, 25 mM MES pH 5.5, 5 mM DTT, 50 mM NaCl. Assays were performed for 5–30 min and fluorescence intensity quantified at Ex 380 nm and Em 450 nm on Envision plate reader. For neutrophil elastase (NE) assays bone marrow lysates were diluted 1:1 with PBS (containing Ca2+ and Mg2+). NE activity in bone marrow lysates was determined in 96-well assay plates (Nunc 475515) containing 50 μl cell lysate and 50 μl of 300 μM N-methoxysuccinyl-Ala-Ala-Pro-Val-AMC (Sigma M9771) in PBS (containing Ca2+ and Mg2+). Assays were performed for 5–30 min and fluorescence intensity quantified at Ex 380 nm and Em 450 nm on Envision plate reader. For cathepsin G (CG) assays bone marrow lysates were diluted 1:20 with 100 mM Tris-HCl pH7.5, 1 M NaCl. CG activity in bone marrow lysates was determined in 96-well assay clear bottomed plates (Costar) containing 50 μl cell lysate and 50 μl of 2 mM N-succinyl-Ala-Ala-Pro-Phe-pNA (Sigma S7388) in 100 mM Tris-HCl pH7.5, 1 M NaCl. Assays were performed for 30 min-4 h and absorbance at 405 nm read on Envision plate reader. Calpain protease activity in BAL samples was quantified using Calpain-Glo luminescent assay (Promega G8501) according to manufacturer’s instructions.

### Biochemical IL-33 assays

Human rFL IL-33 lysate, mock lysate or rIL-33 (112–270 aa) (1800 ng/ml) were incubated with purified human calpain-1 (150 ng/ml), neutrophil elastase (25 ng/ml) or cathepsin G (25 ng/ml) (Calbiochem 208713, 324681, 219373 respectively) for 2–4 h. Assay buffer for calpain-1 was 10 mM Tris-HCl, 150 mM NaCl, 1 mM DTT, 2 mM CaCl2 and for NE and CG was PBS (with Ca2+ and Mg2+). Recombinant mouse and human FL IL-33 lysate, mature IL-33, cysteine mutant IL-33 and DSB-IL-33 (all 112–270 human or 109–266 aa mouse) (1800 ng/ml) were incubated with 300 μg/ml ALT for 1–10 min in PBS (with Ca2+ and Mg2+). In some experiments ALT was pre-incubated with protease inhibitors, other inhibitors, or vehicle for 15 min.

### Epithelial cell IL-33 assays

Mouse alveolar epithelial cell line CMT-64 cells (Cancer Research Technology Ltd, Licence 011671) were supplied by ECACC, Public Health England and were maintained in DMEM/10% FCS according to supplier’s protocols. Normal human bronchial epithelial cells (NHBE) were obtained from Cambrex and maintained in BEGM culture media according to supplier’s protocols (Lonza). Cells were confirmed negative for mycoplasma. Unchallenged cells were washed 2 × PBS and harvested directly into SDS-PAGE buffer containing 2% β-mercaptoethanol. Cells were washed 2 × PBS and incubated in serum-free DMEM. Cell supernatants were collected and cell lysates were harvested by incubation in PBS containing 1% Triton X100 for 30 min on ice, scraping and centrifugation (to remove cell debris). In some experiments cells were pre-incubated with protease inhibitors or other inhibitors and/or treated with 0.1–100 μM ionomycin (Sigma I0634) or 100 μg/ml ALT before harvesting.

### Histological analysis

Histological analysis was performed by standard haematoxylin and eosin staining of formalin fixed, paraffin embedded 10 μm mouse lung tissue sections 1, 6 and 24 h after treatment with 25 μg i.n. ALT or PBS.

### Immunohistochemical staining

Paraffin embedded mouse or human lung tissue sections were subjected to heat induced epitope retrieval (HIER) in a pretreatment module (Dako PT-link, Dako Cytomation, Glostrup, Denmark) before immunostaining in an automated immunohistochemistry robot (AutostainerPlus, Dako). Mouse tissue sections were sequentially blocked with EnVision™ FLEX Peroxidase-Blocking Reagent and serum free protein block (both Dako), incubated with goat anti-mouse IL-33-antibody (1:1000, R&D Systems, Minneapolis) followed by anti-goat-HRP (Dako) and diaminobenzidine (DAB) for visualisation. Human tissue sections were blocked with EnVision™ FLEX Peroxidase-Blocking Reagent, incubated with mouse anti-human IL-33 (1:900, Nessy-1, Axxora), followed by polymer/HRP linked secondary antibodies (Dako) and DAB. Nuclei were counterstained with Mayer’s hematoxylin (blue nuclei), sections dehydrated through ethanol series, cleared in xylene and mounted in Pertex (HistoLab).

For double staining of IL-33 and pro-SPC paraffin embedded mouse lung tissue sections were processed in a pretreatment module and then stained for IL-33 as previously described. Sections were then treated with denaturing solution (Biocare Medical, USA) followed by incubation with anti-pro-SPC antibodies and goat anti-rabbit Alexa488-conjugated antibodies (Life Technologies, Carlsbad, CA, USA). Finally, the tissue was treated with Hoechst dye, and mounted in PBS/glycerol.

### Transmission electron microscopy

Ultrastructural alterations after ALT exposure were performed as described^74^. Briefly, small cubes (1–3 mm^3^) of fresh mouse lung tissue were placed in EM fixative (1% glutaraldehyde and 3% formaldehyde in buffered PBS). Samples were incubated in 1% osmium tetroxide for 1 h, dehydrated and embedded in Epon 812 resin. Toluidine blue-stained plastic sections (1 μm) of whole samples were used to select areas for detailed ultrastructural analysis. Ultra-thin sections (60 nm) were prepared from the selected areas using an ultratome and a diamond knife, contrast stained with metal salts (osmium and lead citrate) and placed in 200-mesh copper grids. Ultrastructural analysis was performed with a Phillips/Fei transmission electron microscope.

### SDS-PAGE and western blot analysis

Samples were analysed by SDS-PAGE on NuPAGE Novex 4–12% or 12% Bis-Tris mini gels (Invitrogen) with MOPS running buffer (Invitrogen) according to manufacturer’s instructions under reducing conditions. All samples were reduced by heated to 95 C for 3 min in SDS-PAGE buffer containing 2% beta-mercaptoethanol. Gels were stained with Coomassie brilliant blue G-250 based InstantBlue gel staining (Sigma). For some studies, proteins were transferred to nitrocellulose membranes (Invitrogen) and detected by Western blotting. IL-33 was detected with goat anti-IL-33 pAb (R&D systems AF3625, AF3626) at 1:1000 dilution^17^. Calpain-1 and -2 were detected with rabbit anti-calpain-1 or -2 pAb (Abcam ab28257, ab39165) respectively at 1:1000 dilution^50,75^. Immunoreactive proteins were identified with HRP-conjugated anti-goat or anti-rabbit (R&D systems HAF109 and HAF008 respectively) and Supersignal West Femto substrate (Pierce 34095).

### Immunoprecipitation experiments

Human anti-IL-33 mAb (#H338L293 and #640050) and mAb isotype controls (NIP228) were biotinylated via free amines using EZ link Sulfo-NHS-LC-Biotin (Thermo/Pierce) or via free cysteines using EZ link Biotin-BMCC (Perbio/Pierce) and conjugated to Streptavidin-coated beads (Invitrogen, M-280 Dynabeads) according to manufacturer’s instructions. Mouse rIL-33 (109–266 aa, 10 ng/ml) or 500 μl BAL from mice, 30 min after ALT or PBS challenged mice, were incubated with 2.5 μg bead-conjugated mAb (H338L293 or NIP228) for 1–2 h in 1 ml of 20 mM Tris-HCl pH 7.5, 150 mM NaCl, 1.5 mM CaCl2, 1.5 mM MgCl2, 0.1% Triton X100, 0.1% octylglucoside, 0.1% CHAPS, 5% glycerol, 0.1% BSA. Beads were collected, washed in 5 × 1 ml of IP buffer without BSA, and proteins eluted by boiling for 5 min in SDS-PAGE buffer containing 2% β-mercaptoethanol. Human rIL-33 (112–270 aa, 10 ng/ml), 500 μl human lung lysate or 500 μl human lung explant supernatant was incubated with 2.5 μg bead-conjugated mAb (#640050 or NIP228) and IP performed as described for mouse experiments.

### Cell culture and bioassays

Normal human umbilical vein endothelial cells (HUVECs) were obtained from Cambrex and maintained in EBM-2 culture media (Lonza) according to manufacturer’s protocol. Cells were confirmed negative for mycoplasma. For signalling assays cells were stimulated with IL-33 for 30 minutes, fixed with formaldehyde and stained for NFkB p65 subunit (Thermo-Fisher) according to manufacturer’s protocol. Hoechst dye was used to stain the cell nuclei. Plates were analysed for nuclear NFkB p65 translocation using an ArrayScan VTi HCS Reader. For cytokine assays cells were stimulated with IL-33 for 20 h and supernatants analysed for levels of IL-6 and IL-8 by Duoset ELISA (R&D systems DY206, DY208) according to manufacturer’s protocol. Data were analysed using Graphpad Prism software. IC_50_ values were determined by curve fitting using a three or four-parameter logistic equation.

### Cell cytotoxicity

Lactate dehydrogenase (LDH) enzymatic activity was measured using CytoTox 96 non-radioactive cytotoxicity assay according to manufacturer’s instructions (Promega G1780).

### Immunocytochemistry

CMT-64 cells were seeded on collagen coated 96 well plates (Greiner) for 24 h then fixed with formaldehyde. CMT-64 cells were stained using Nessy-1 mAb (ALX-804–840, Enzo Life Sciences) or mouse IgG1 isotype control mAb (ADI-SAD-600, Enzo Life Sciences). DAPI (D1306, Life Technologies) was used to counterstain the cell nuclei. An Alexa Fluor-488 conjugated anti-mouse mAb (A11001, Life Technologies) was used to visualise staining. Plates were analysed using the ArrayScan VTi HCS Reader (NHBE) at 10× magnification, or the ImageXpress Micro XLS (CMT-64) at 20× magnification.

### Statistical analyses

All data are expressed as means ± SEM. GraphPad Prism (GraphPad Software Inc., La Jolla, CA, USA) were used for one way and two way ANOVA statistical analyses.

## Electronic supplementary material

Supplementary Information
