# Supplementary material for: Interleukin-33 is activated by allergen- and necrosis-associated proteolytic activities to regulate its alarmin activity during epithelial damage
Source: Sci Rep. 2018 Feb 20;8:3363. doi: 10.1038/s41598-018-21589-2 (PMC5820248; doi:10.1038/s41598-018-21589-2)
Supplement: Supplementary file 1 — Supplementary Information [file 41598_2018_21589_MOESM1_ESM.pdf]

Supplementary Information

Interleukin-33 is activated by allergen- and necrosis-associated proteolytic activities to regulate its alarmin activity during epithelial damage

Ian C. Scott[1], Jayesh B. Majithiya[1], Caroline Sanden[3], Peter Thornton[2], Philip Sanders[1], Tom Moore[1], Molly Guscott[1], Dominic J. Corkill[1], Jonas S. Erjefält[3], E. Suzanne Cohen[1]

Supplementary figure 1

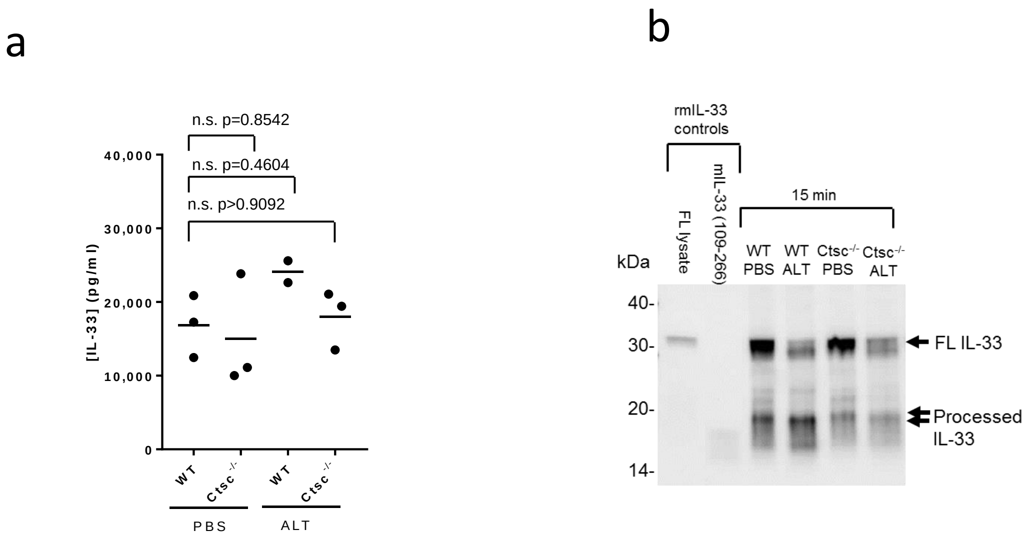

Supplementary Figure 1:

(**a**) Concentration of IL-33 (pg/ml) in WT and *Ctsc*$^{-/-}$ mouse lung lysates 30 min after i.n. ALT or PBS challenge (n=3-4/mice group). Data points are mean ± SEM. Statistical analysis: one way ANOVA, Tukey's post-test, F=1.64, degrees of freedom=3. n.s.: non-significant between all groups. (**b**) Western blot of IL-33 in lung lysates from WT and *Ctsc*$^{-/-}$ mice 15 min after i.n. ALT or PBS challenge (n=3-4 pooled/group). Controls as: FL lysate, lysate of CHO cells transfected with full length mouse IL-33; mIL-33 (109-266), recombinant mouse IL-33 109-266 aa. Representative of n=3 independent experiments (**a, b**).

Supplementary figure 2

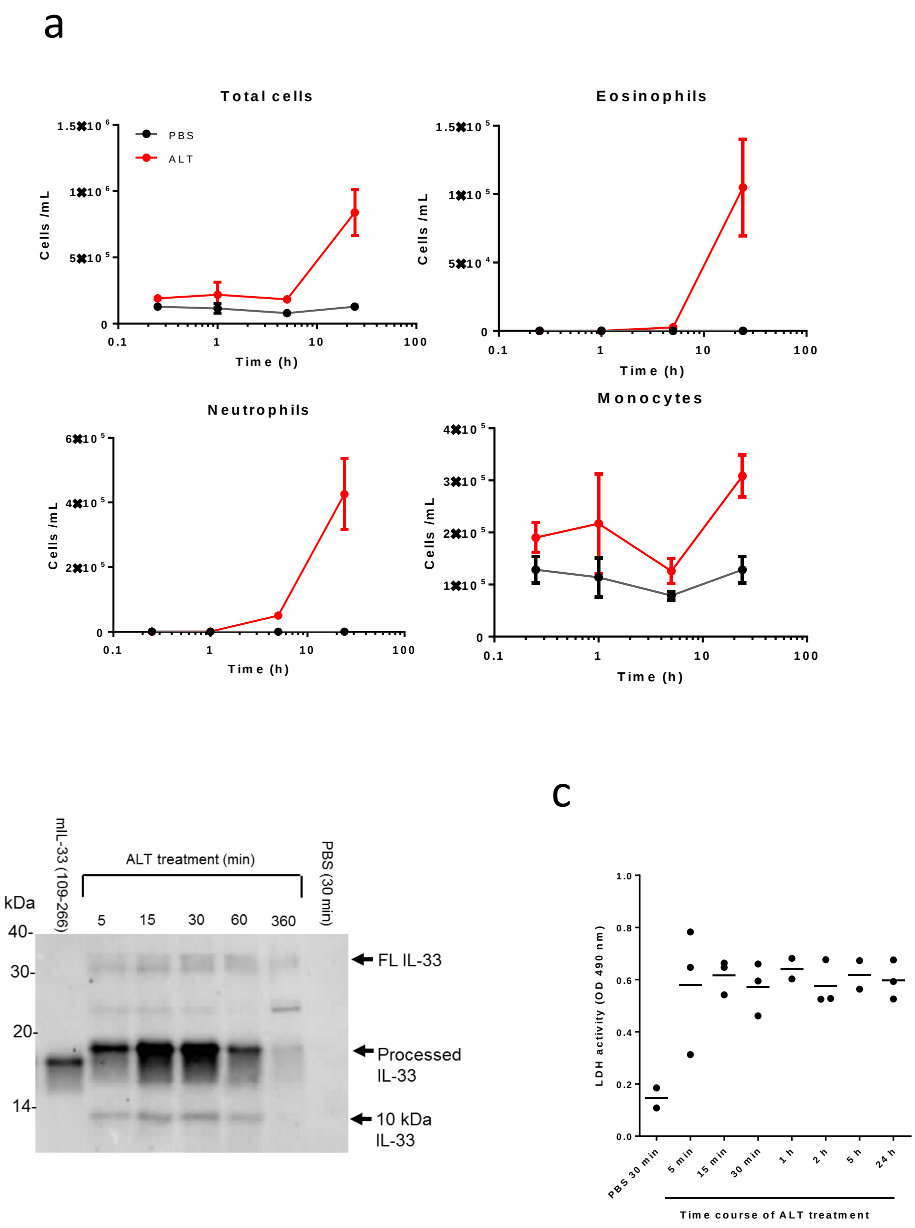

Supplementary Figure 2:

(**a**) Differential cell counts (cells/ml) in BAL 15 min to 24 h following i.n. ALT or PBS challenge (n=12 mice/group). Pooled analysis of n=3 experiments. (**b**) Western blot of IL-33 in BAL (pooled n=3 mice/group) 5 min to 6 h after ALT or PBS challenge. Controls: mIL-33 (109-266), recombinant mouse IL-33 (109-266 aa). (**c**) Lactate dehydrogenase activity (n=2-3 mice/group) in BAL 5 min to 24 h after ALT or PBS challenge. Representative of n=3 independent experiments (**b**) and n=2 independent experiments (**c**).

Supplementary figure 3

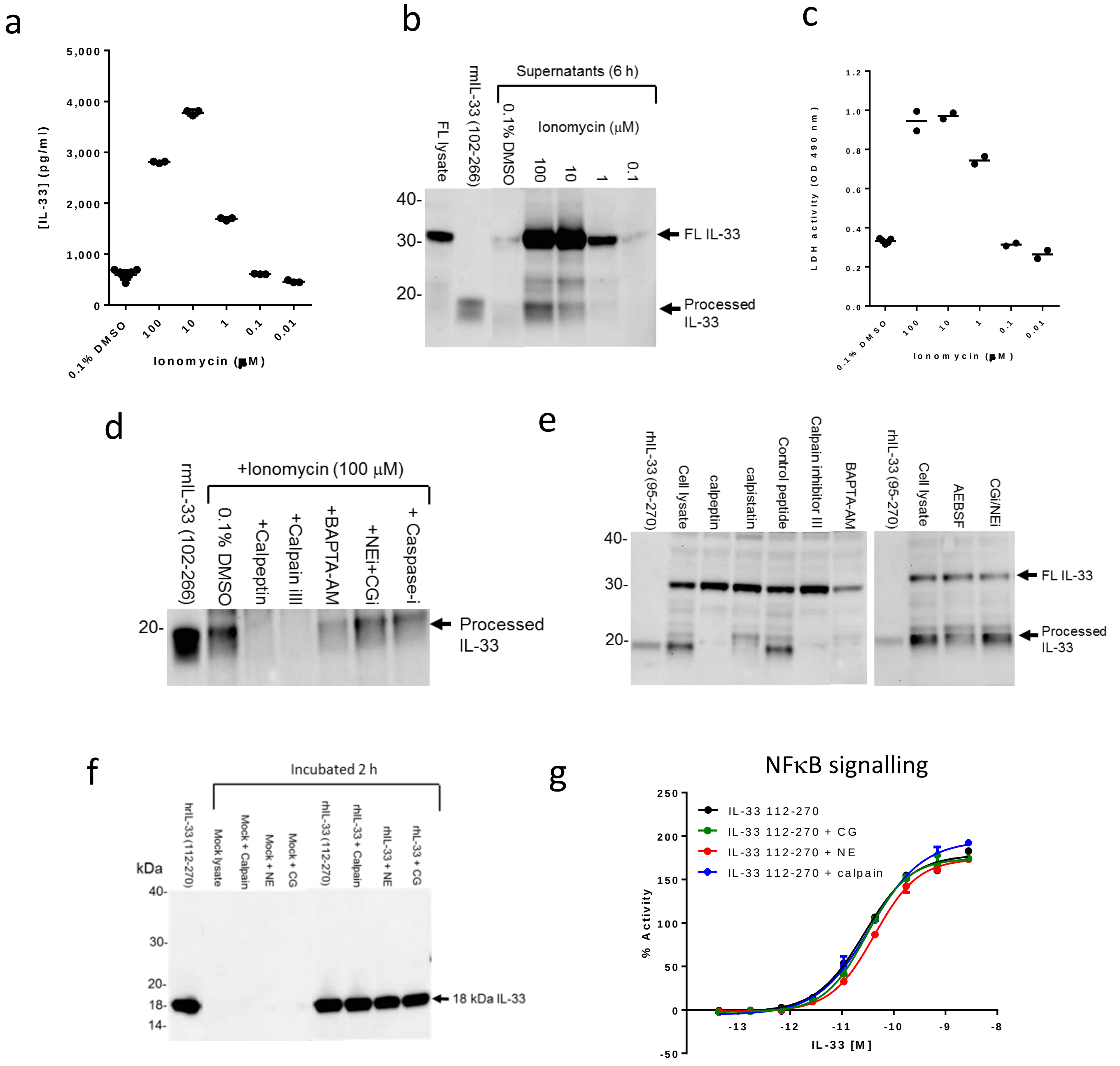

Supplementary Figure 3:

(**a**) The concentration of IL-33 (pg/ml) in CMT-64 cell supernatants after incubation for 6 h with ionomycin or 0.1% DMSO. (**b**) Western blot of mouse IL-33 in CMT-64 cell supernatants (as a). Controls: FL lysate, lysate of CHO cells transfected with full length mouse IL-33; rmIL-33 (102-266), mouse IL-33 (102-266 aa). (**c**) Lactate dehydrogenase activity of CMT-64 cell supernatants (from a, b). (**d**) Western blot of mouse IL-33 in CMT-64 cell supernatants. Gel image is cropped to show only processed IL-33. Cells were pre-treated for 30 min with protease inhibitors, BAPTA-AM or 0.1% DMSO, with addition of ionomycin for 2 h. Controls are as (b). (**e**) Western blot of IL-33 in NHBE lysates. Cells were pre-treated for 30 min with protease inhibitors, or 0.1% DMSO (cell lysate), and incubated for 30 min in PBS/0.1% Triton X100. Controls: rhIL-33 (95-270), purified recombinant human IL-33 (95-270 aa). (**f**) Western blot of mature rhIL-33 (112-270) and mock cell lysates that were incubated alone or with calpain, NE and CG for 2 h. Controls: rhIL-33, recombinant human IL-33, 112-270, purified recombinant human IL-33 (112-270), Mock, lysate from mock transfected HEK cells. (**g**) Relative levels of NFkB p65/RelA translocation in HUVECs 30 min after stimulation by rhIL-33 (112-270) pre-incubated alone or with calpain, NE and CG for 2 h.. Data points are mean ± SEM of duplicate determinations. %Activity is calculated relative to signal of 3 ng/ml rhIL-33 (112-270). (**h**) Representative of n=3 independent experiments (**d, f, g**) and representative of n=2 independent experiments (**a-c, e**)

Supplementary figure 4

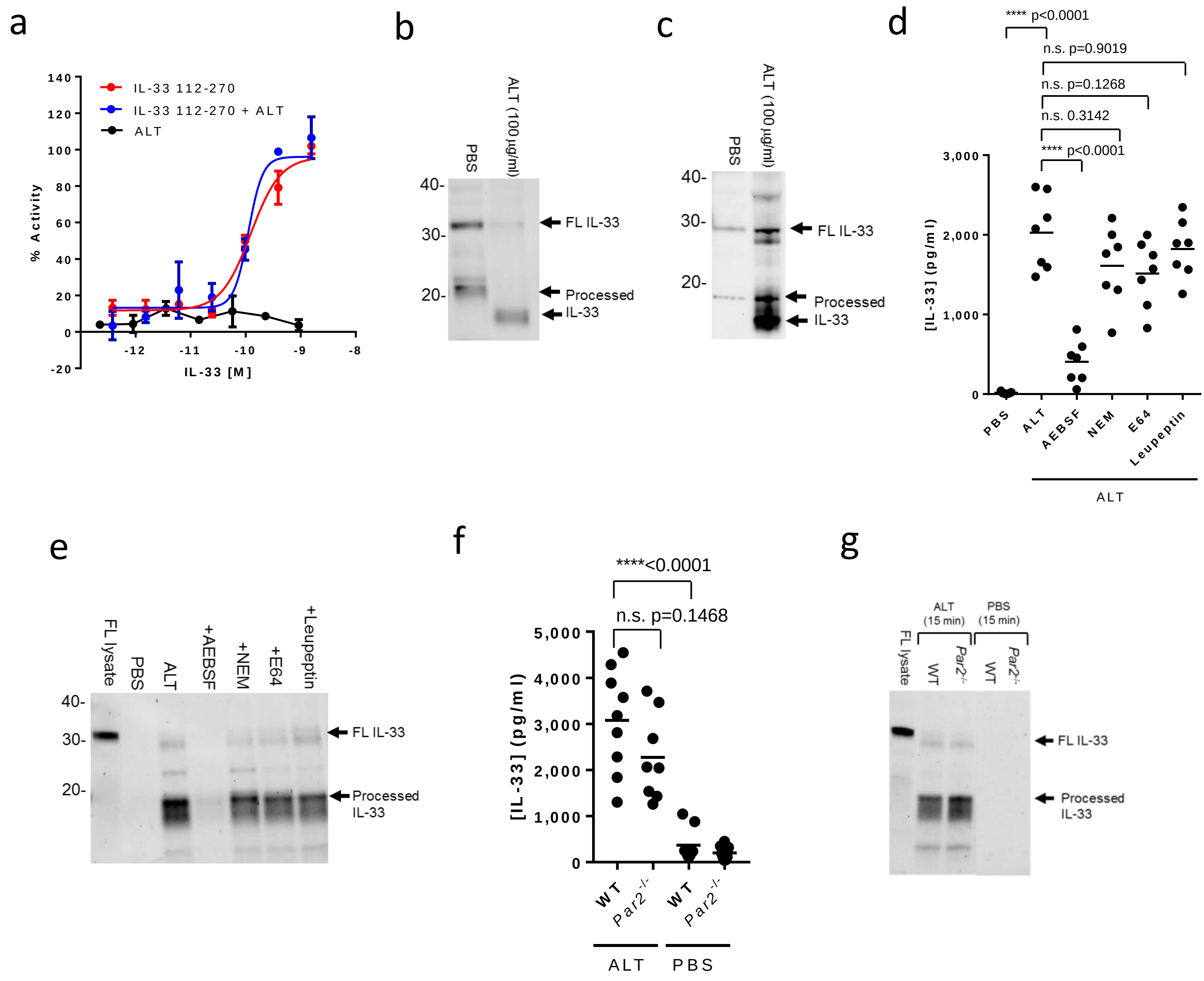

Supplementary Figure 4:

(**a**) Relative levels of NFκB p65/RelA translocation in HUVECs 30 min after stimulation by rhIL-33 (112-270) incubated for 10 min with or without 300 µg/ml ALT and 300 µg ALT alone. %Activity is calculated relative to signal of 3 ng/ml rhIL-33 (112-270). (**b**) Western blot of human IL-33 in NHBE cell lysates 30 min after 100 µg/ml ALT or PBS challenge. (**c**) Western blot analysis of human IL-33 in NHBE cell supernatants 30 min after 100 µg/ml ALT or PBS challenge. (**d**) Concentration of IL-33 (pg/ml) in BAL 15 min after ALT or PBS challenge with and without co-administration of protease inhibitors (AEBSF, NEM, E64 and leupeptin). Statistical analysis: 1-way ANOVA test, Tukey's post test, F=33.8, degrees of freedom=5. n.s.: non-significant (**e**) Western blot of mouse IL-33 in BAL 15 min after ALT or PBS challenge with and without co-administration of protease inhibitors (AEBSF, NEM, E64 and leupeptin). Controls: FL lysate, lysate of CHO cells transfected with FL mouse IL-33. (**f**) Concentration of IL-33 (pg/ml) in BAL 15 min after ALT or PBS challenge of WT and *Par2*$^{-/-}$ mice (pooled n=3 mice/group). Statistical analysis: 1-way ANOVA test, Tukey's post test, F=30.7, degrees of freedom=3. n.s.: non-significant (**g**) Western blot of mouse IL-33 in BAL 15 min after ALT or PBS challenge of WT and *Par2*$^{-/-}$ mice (pooled n=3 mice/group). Controls as (e). Data is pooled from n=3 independent studies (**d, f**). Representative of n=3 independent experiments. (**a, e**) and representative of n=2 independent experiments (**b, c, g**).

Supplementary figure 5

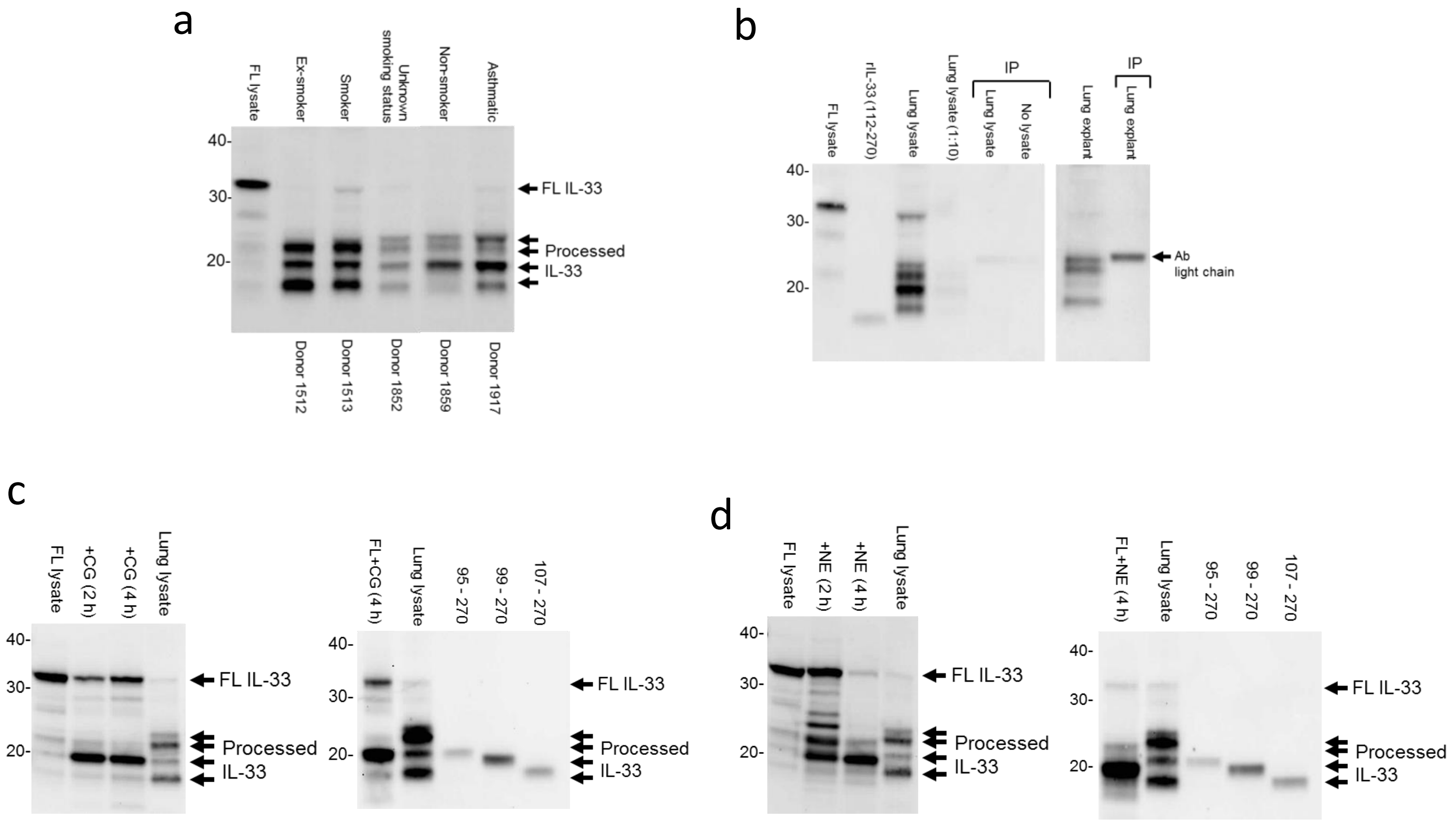

Supplementary Figure 5:

(**a**) Western blot of IL-33 expression in human lung tissue lysate from ex-smoker (donor 1512), smoker (1513), unknown smoking status (1852), non-smoker (1859) and asthmatic (1917) patients. Controls are as follows: FL IL-33, lysate of HEK cells transfected with full length human IL-33. (**b**) Immunoprecipitation (IP) and western blot of IL-33 in human lung lysate and explant supernatants (2 h). IP was performed using a human isotype mAb (NIP228) and western blot with anti-IL-33 pAb (AF3626). Controls: FL lysate, lysate of HEK cells transfected with full length human IL-33; rhIL-33 (112-270), recombinant human IL-33 (112-270 aa). (**c**) Western blot of rhFL IL-33 lysate, with and without pre-incubation for 2-4 h with CG and human lung lysate (left panel). Western blot of rhFL IL-33 lysate, pre-incubated for 4 h with CG, and rhIL-33 95-, 99- and 107-270 aa (right panel). (**d**) Western blot of rhFL IL-33 lysate, with and without pre-incubation for 2-4 h with NE and human lung lysate (left panel). Western blot of rhFL IL-33 lysate, pre-incubated for 4 h with NE, and rhIL-33 95-, 99- and 107-270 aa (right panel). Representative of n=3 (**a**) and n=2 independent experiments (**b-d**).

Supplementary figure 6

Fig. 1e

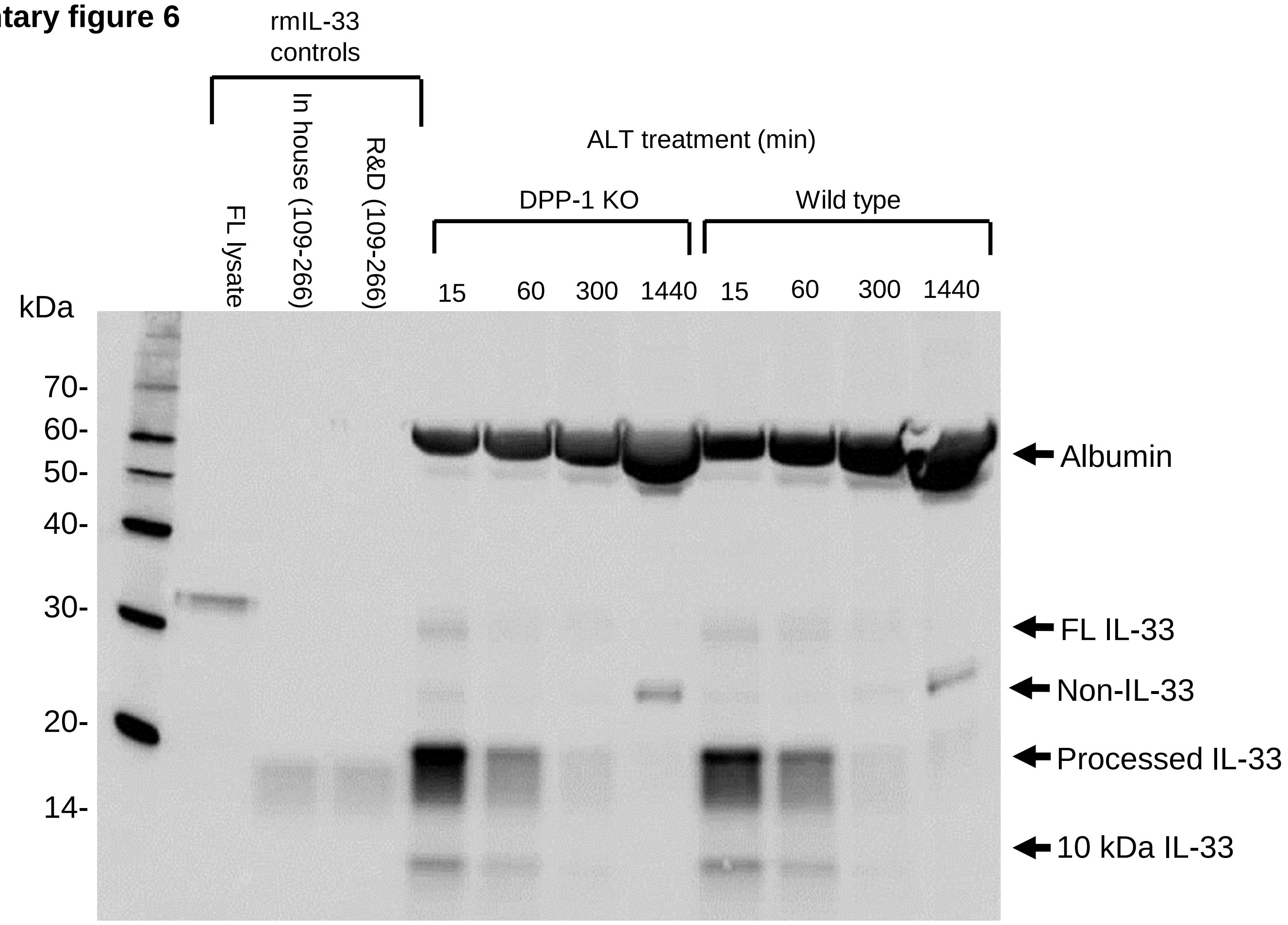

Fig. 1g

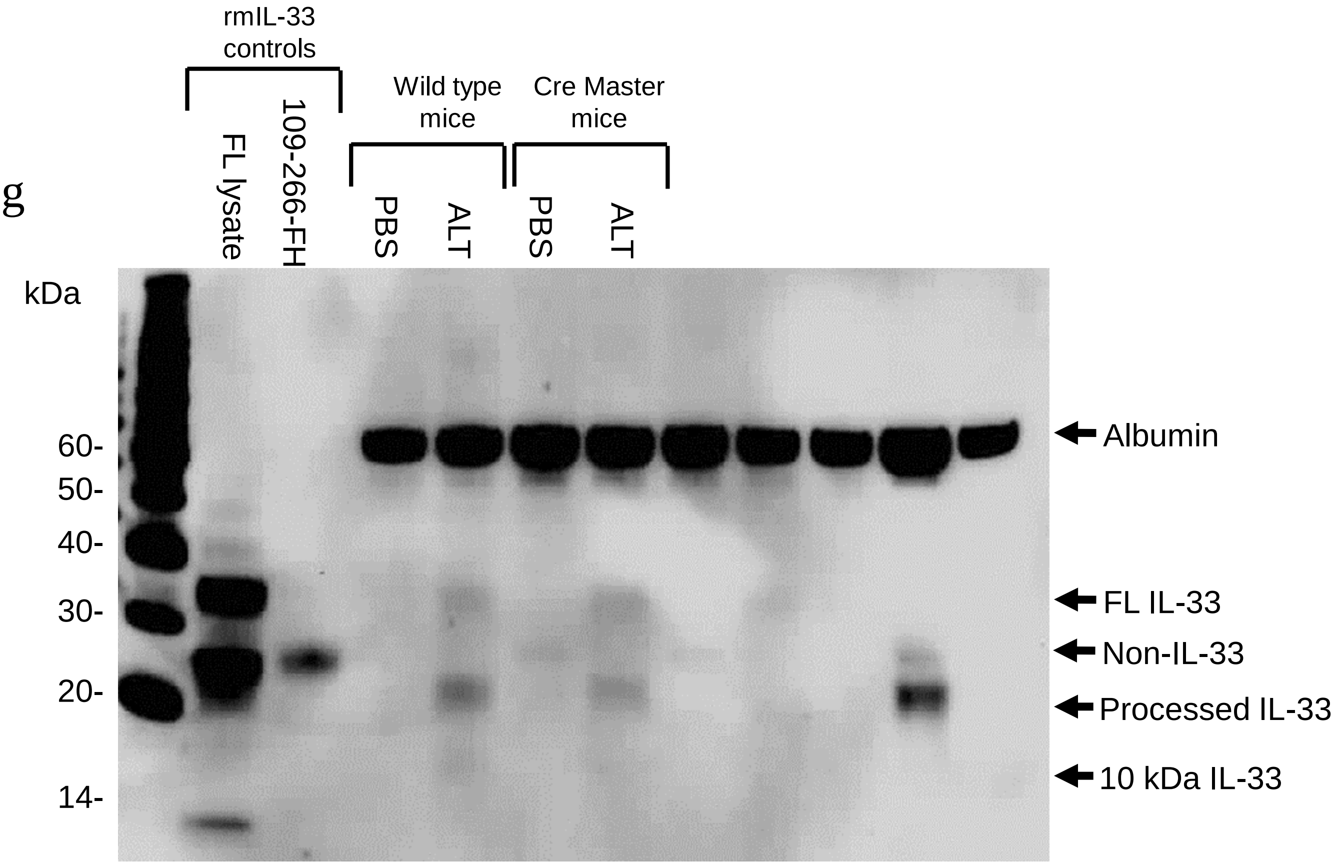

Supplementary Figure 6 – Full blots from Figure 1e and g

(**e**) Western blot of IL-33 of BAL samples (n=3-4 pooled /group). Controls are as follows: FL lysate, lysate of CHO cells transfected with full length mouse IL-33; mIL-33 (109-266), mouse IL-33 (109-266 aa). (**g**) Western blot of IL-33 in BAL samples (n=3-4 pooled /group) from WT and mast cell-deficient mice 30 min after ALT or PBS challenge. Controls: FL lysate, lysate of CHO cells transfected with full length mouse IL-33; 109-266-FH, recombinant mouse IL-33 109-266 aa with N-terminal Flag-His tag.

Supplementary figure 7

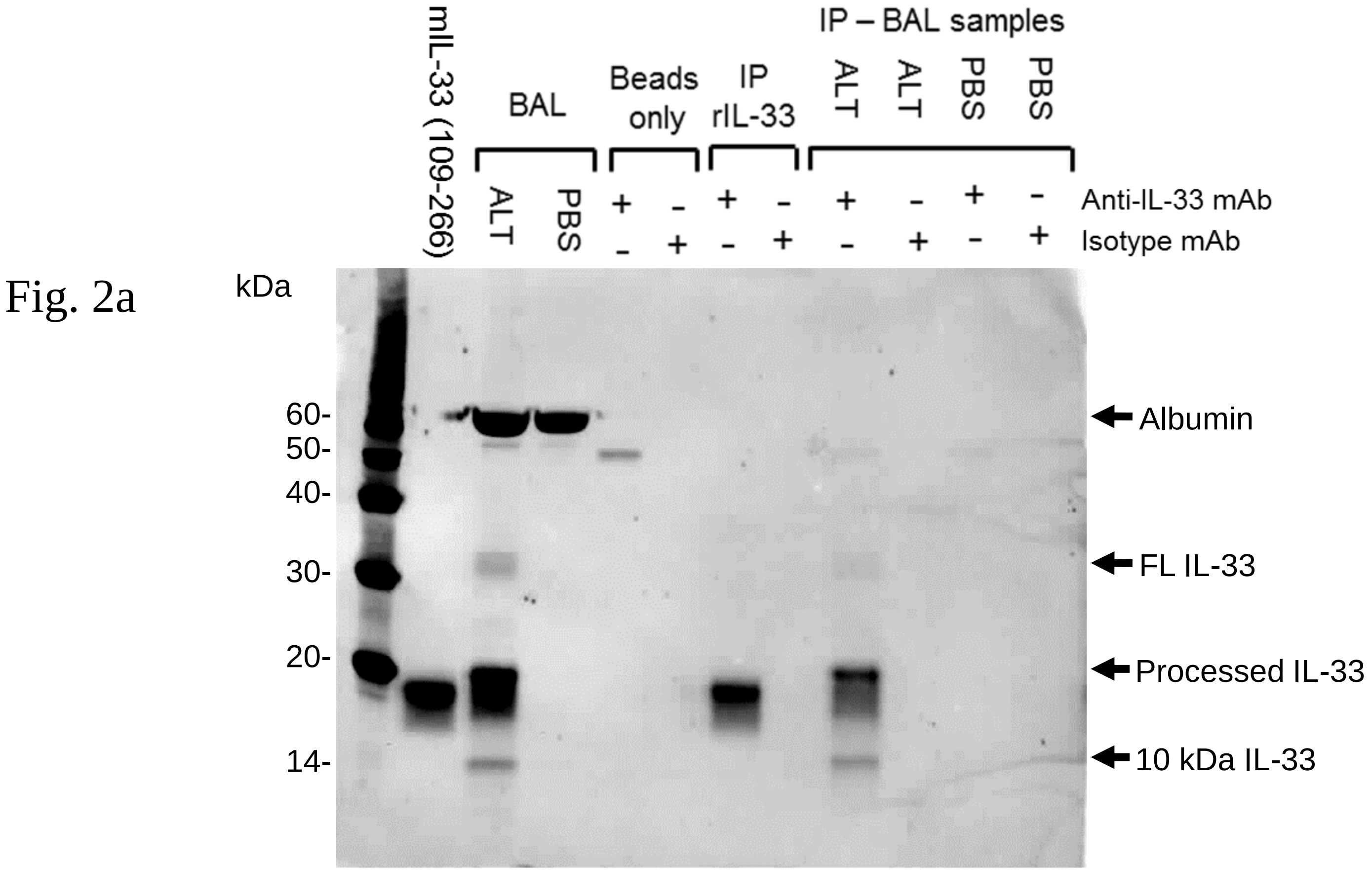

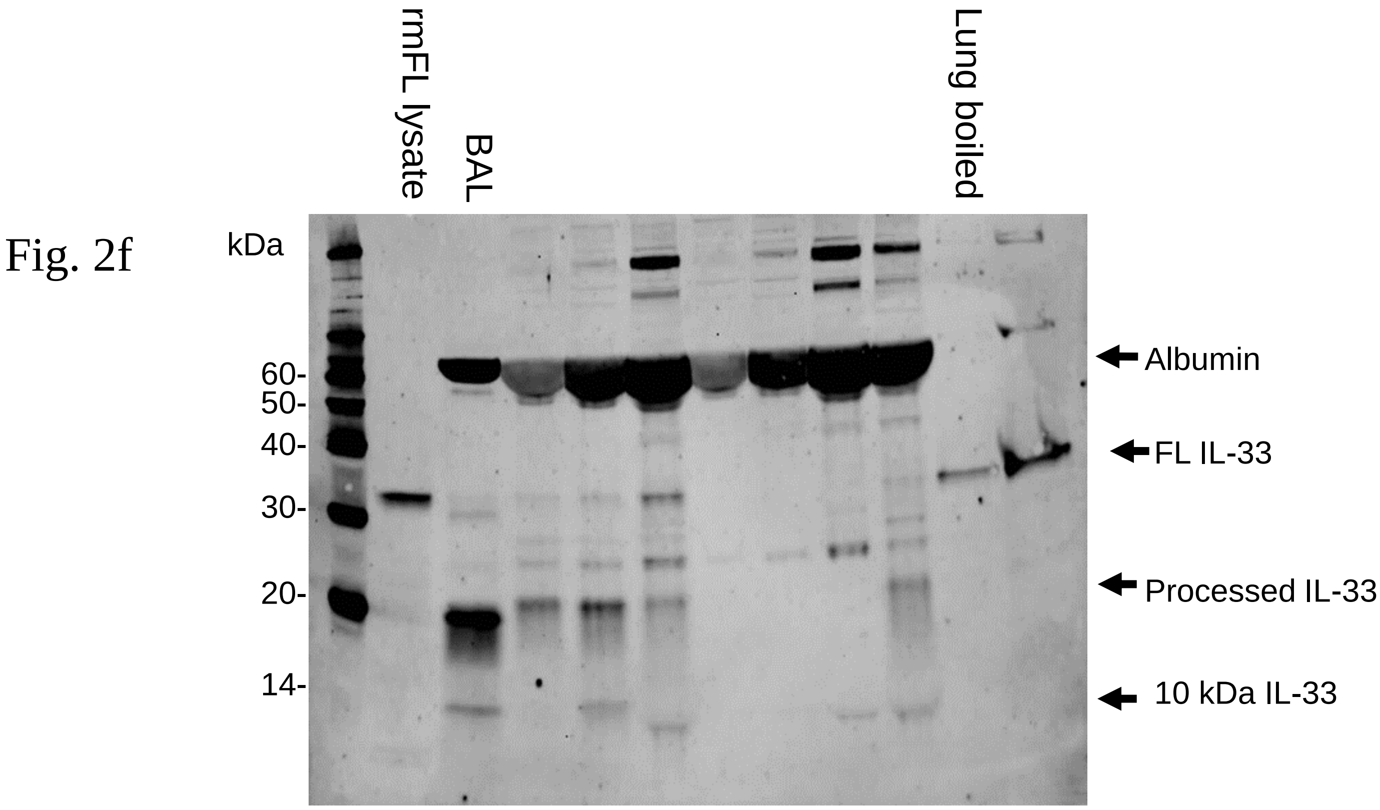

Supplementary Figure 7 – Full blots from Figure 2a and f

(**a**) Immunoprecipitation (#H338L293 or NIP228 mAb) and western blot (AF3626 mAb) of IL-33 in BAL (pooled n=3-4 mice/group) after ALT or PBS challenge. Controls: mIL-33 (109-266), recombinant mouse IL-33 (109-266 aa). Abbreviations: IP, immunoprecipitation. (**f**) Western blot of IL-33 in BAL (pooled n=3 mice) 15 min after ALT challenge and boiled mouse lung (pooled n=3 mice). Controls: FL lysate, lysate of CHO cells transfected with full length mouse IL-33.

Supplementary figure 8

Fig. 3b

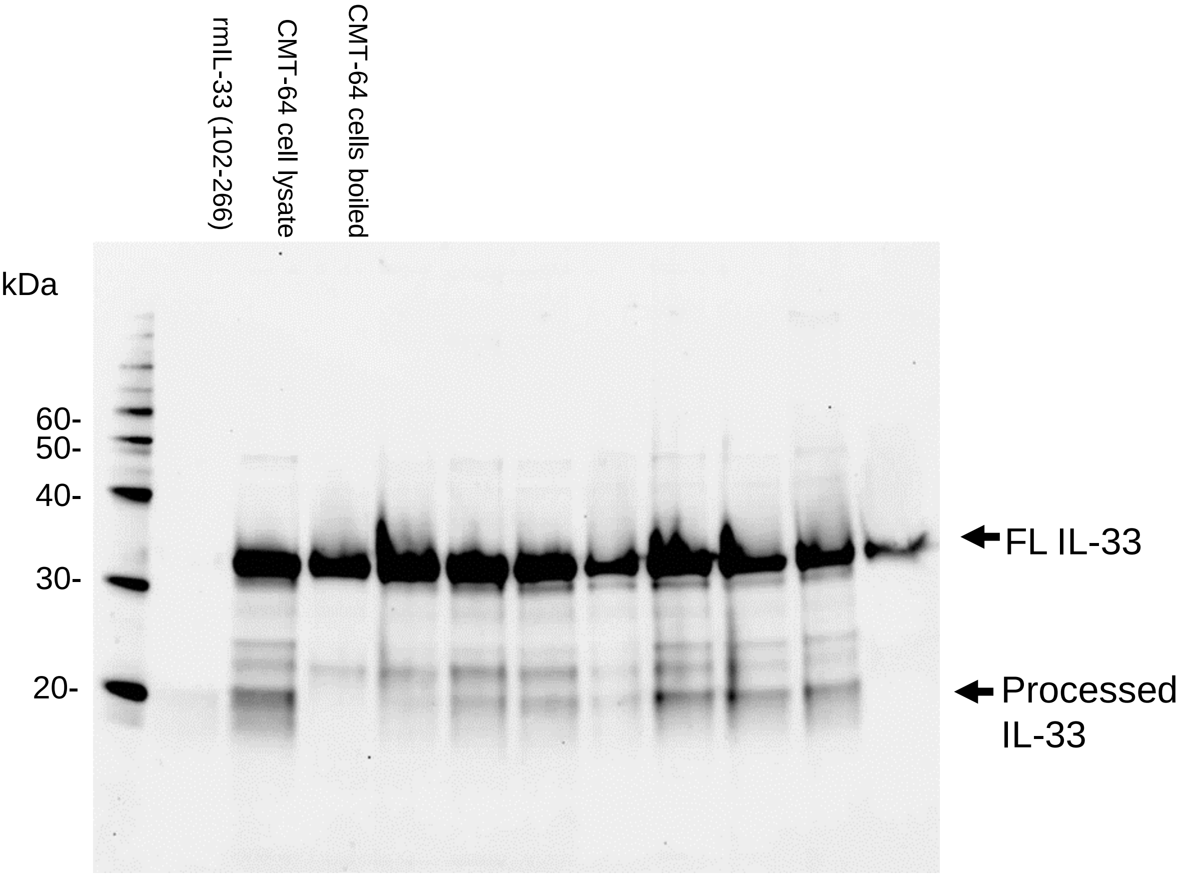

Fig. 3c

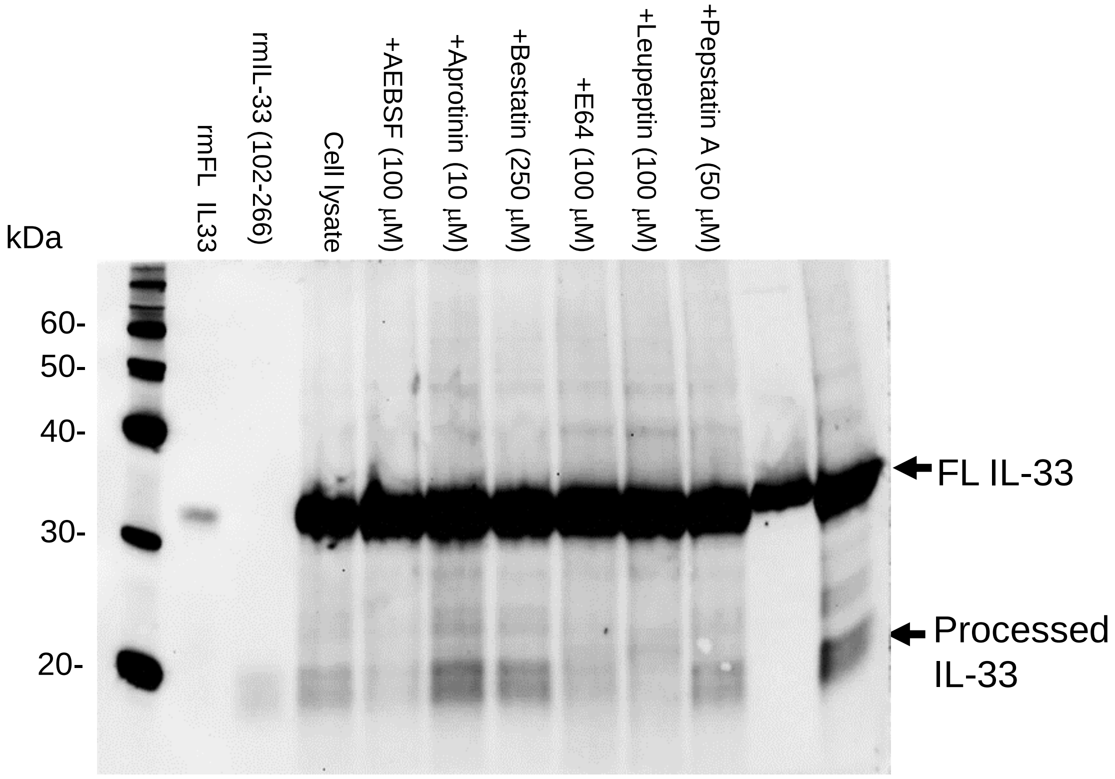

Supplementary Figure 8 – Full blots of Figure 3b and c

(**b**) Western blot of mouse IL-33 in CMT-64 cell lysates (PBS, 0.1% Triton X100) and CMT-64 cells (boiled in SDS-PAGE buffer). Controls: rmIL-33 (102-266), recombinant mouse IL-33 (102-266 aa). (**c**) Western blot of mouse IL-33 in CMT-64 cell lysates. Cells were pre-treated for 30 min with protease inhibitors and incubated and in PBS/0.1% Triton X100 for 30 min. Controls: as (b).

Supplementary figure 9

Fig. 3d

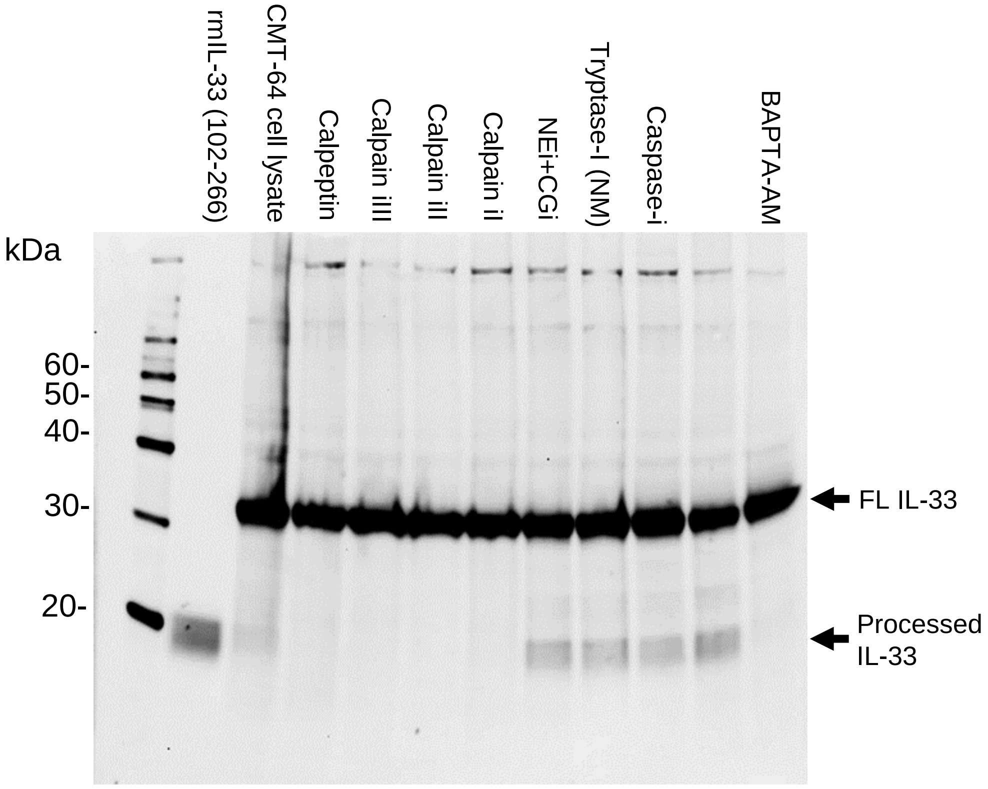

Fig. 3e

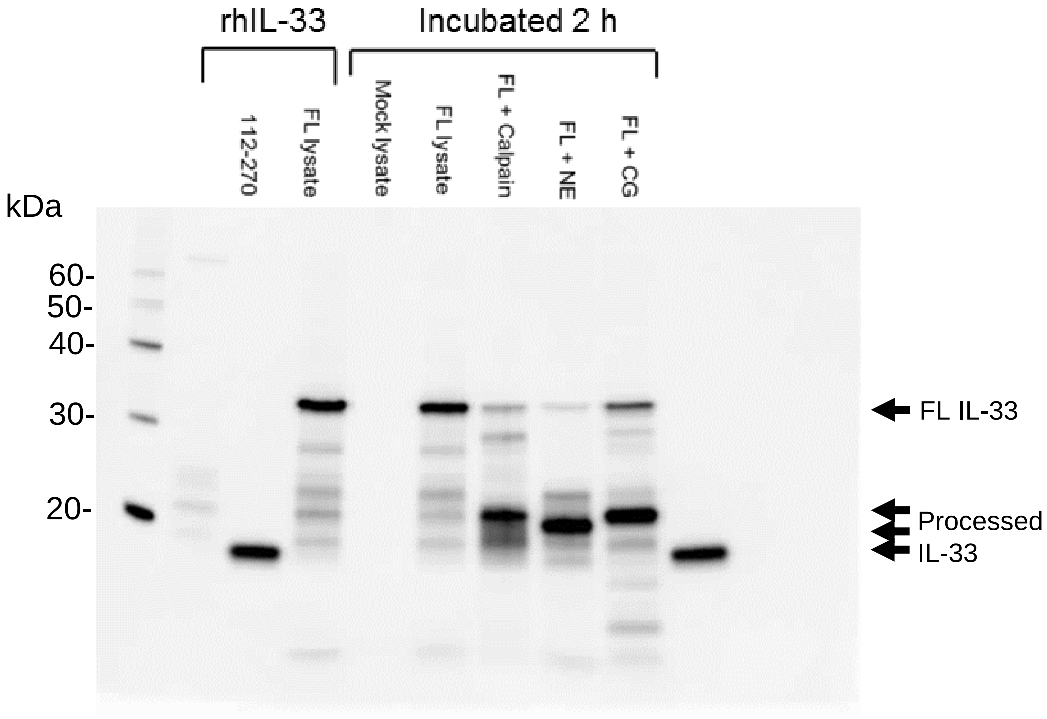

Supplementary Figure 9 – Full blots of Figure 3d and e

(**d**) Western blot of mouse IL-33 in CMT-64 cell lysates. Cells were pre-treated for 30 min with protease inhibitors, BAPTA-AM or 0.1% DMSO (cell lysate) and incubated for 30 min in PBS/0.1% Triton X100. Controls as Fig.3b. Abbreviations: i, inhibitor, NM, nafamostat mesylate. (**e**) Western blot of FL human IL-33 lysate incubated alone or with calpain-1, NE and CG for 2 h. Controls: rhIL-33, recombinant human IL-33, 112-270, purified recombinant human IL-33 (112-270 aa); FL lysate, lysate of HEK cells transfected with full length human IL-33; Mock lysate, lysate of mock transfected HEK cells.

Supplementary figure 10

Fig. 4a
Upper panel

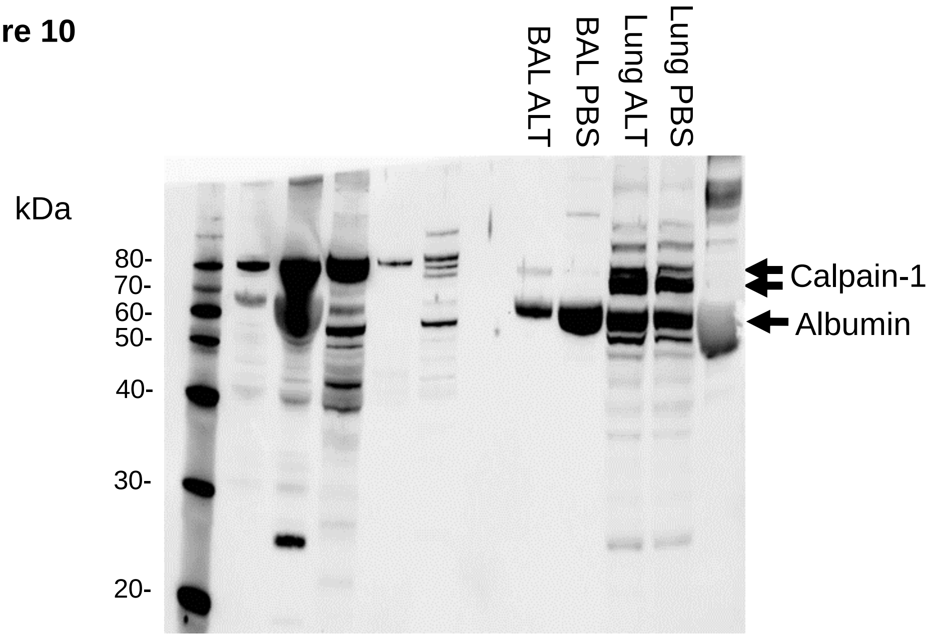

Fig. 4a
Lower panel

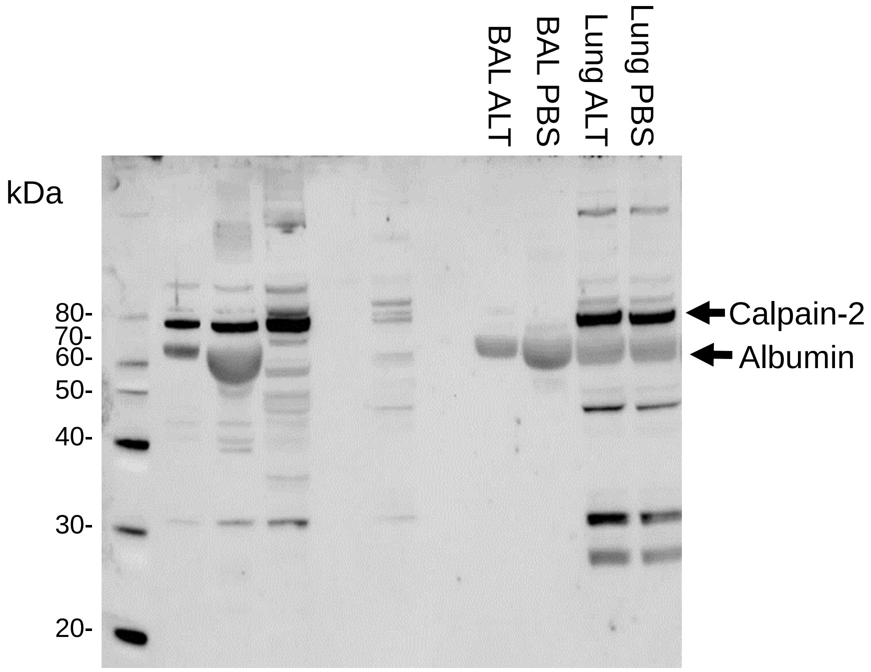

Supplementary Figure 10 – Full blots of Figure 4a

(**a**) Western blot of calpain-1 (upper panel) and -2 (lower panel) in mouse lung homogenates and BAL (pooled n=3-4 mice/group) 30 min after ALT or PBS challenge.

Supplementary figure 11

Fig. 4c

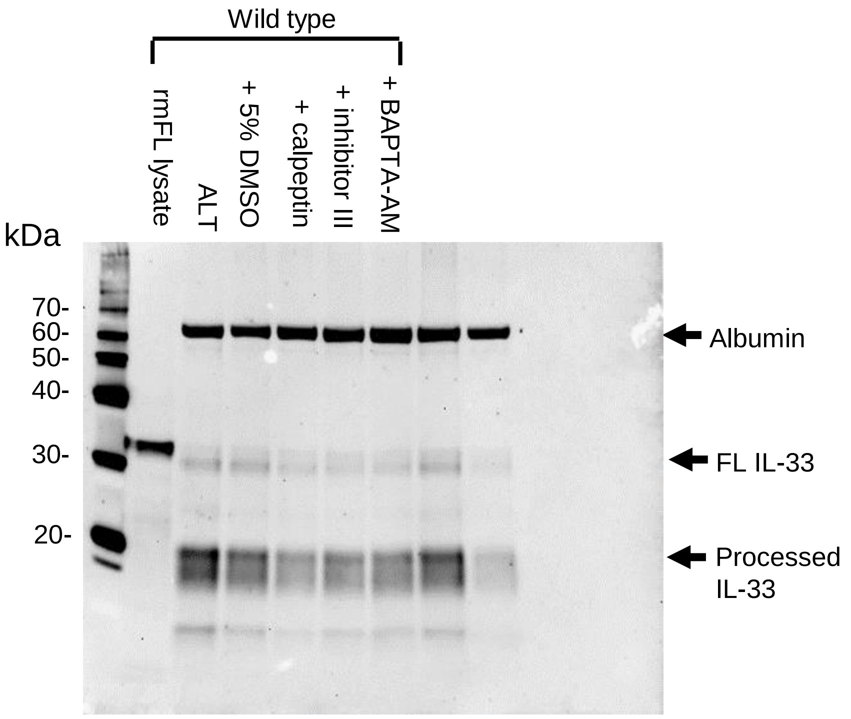

Supplementary Figure 11 – Full blots of Figure 4c

(**c**) Western blot of IL-33 in BAL (pooled n=3-4 mice/group) 15 min after ALT challenge with and without co-administration of calpeptin, calpain inhibitor III, BAPTA-AM or 5% DMSO. Controls: FL lysate, lysate of CHO cells transfected with full length mouse IL-33.

Supplementary figure 12

Fig. 5a

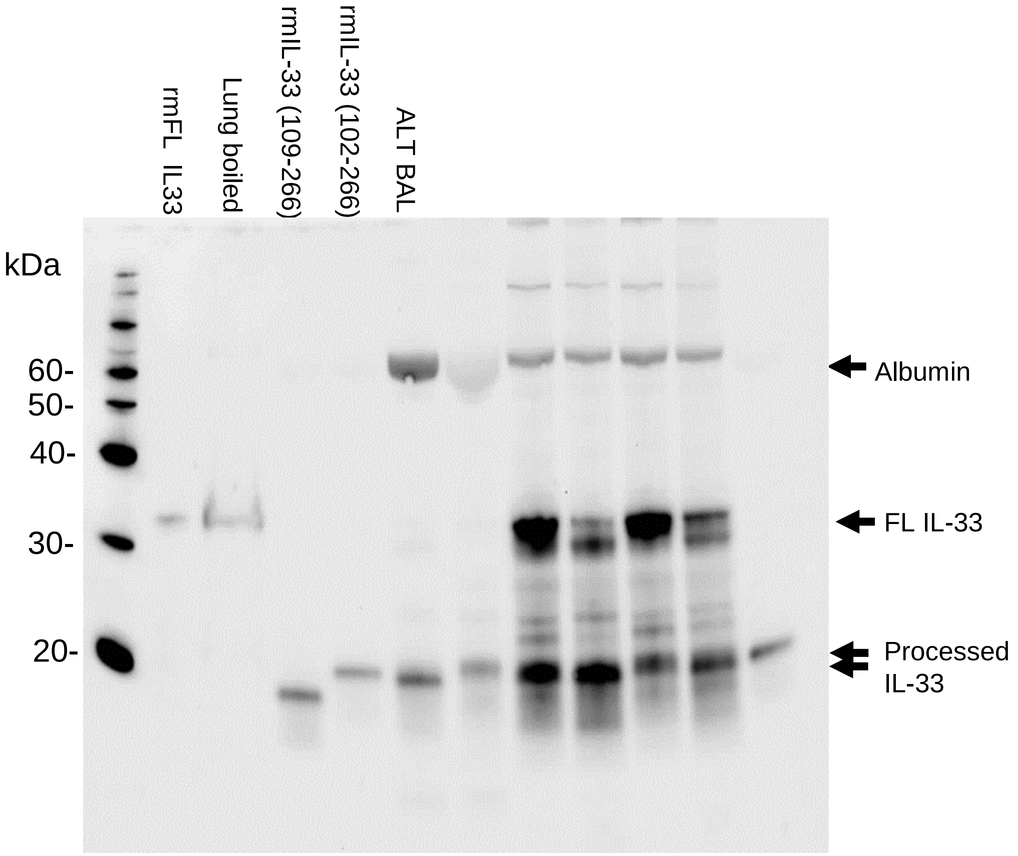

Fig. 5b

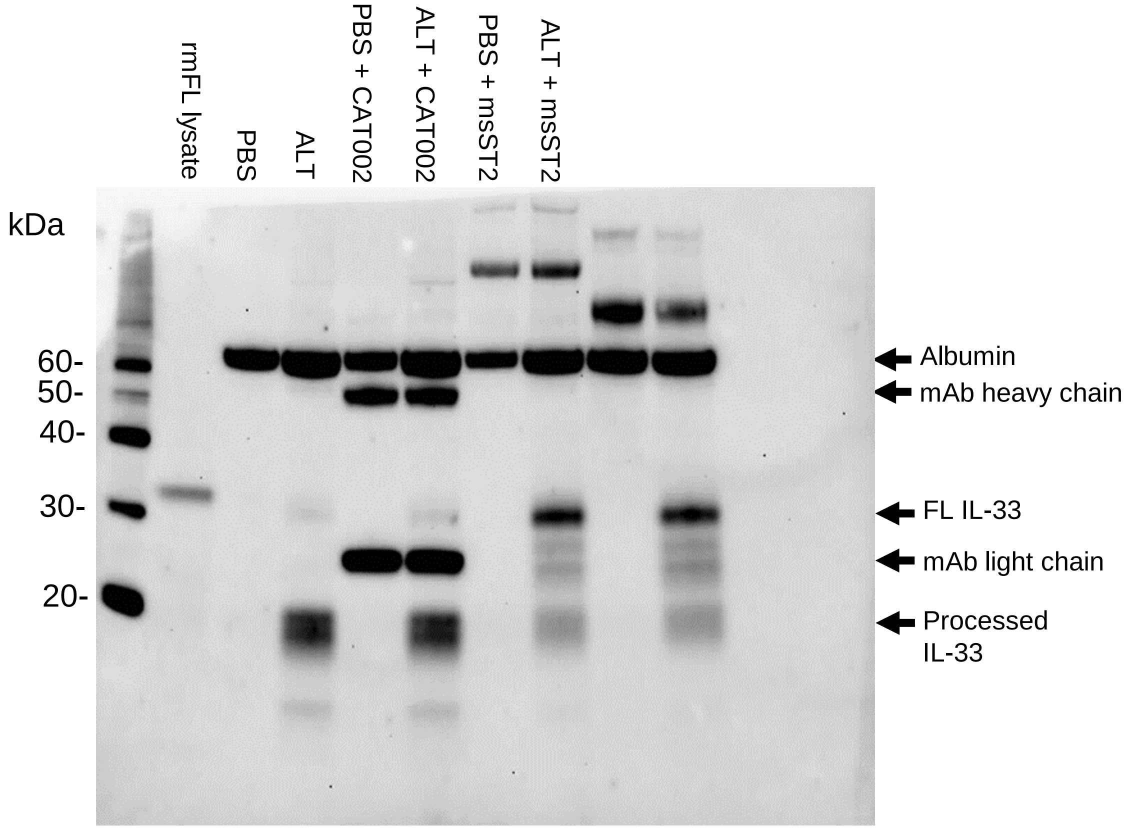

Supplementary Figure 12 – Full blots of Figure 5a and b

(**a**) Western blot of IL-33 in mouse lung (boiled in SDS-PAGE buffer) and BAL 15 min after ALT challenge. Controls: FL IL-33, lysate of CHO cells transfected with full length mouse IL-33; rmIL-33 (102-266 and 109-266), recombinant mouse IL-33 (102- and 109-266 aa). (**b**) Western blot of IL-33 in BAL 15 min after ALT or PBS challenge. Mice were dosed i.p with recombinant mouse sST2-Fc or CAT-002 (control) for 30 min prior to i.n. ALT or PBS challenge and BAL collected after 15 min. Controls as (a).

Supplementary figure 13

Fig. 6c
Upper panel

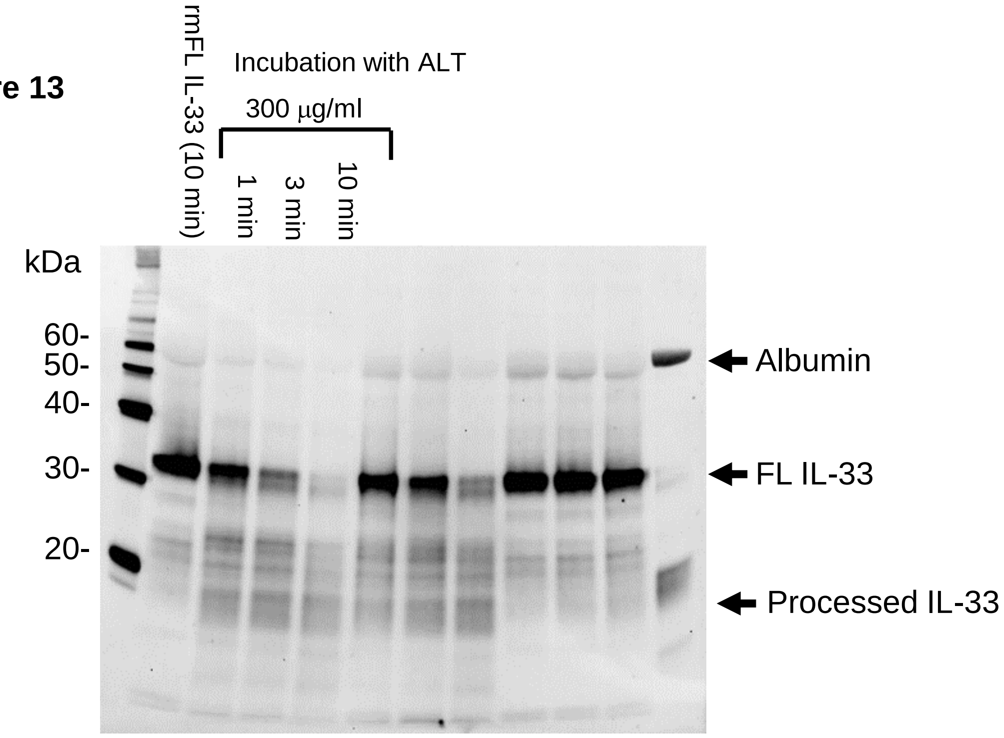

Fig. 6c
Middle panel

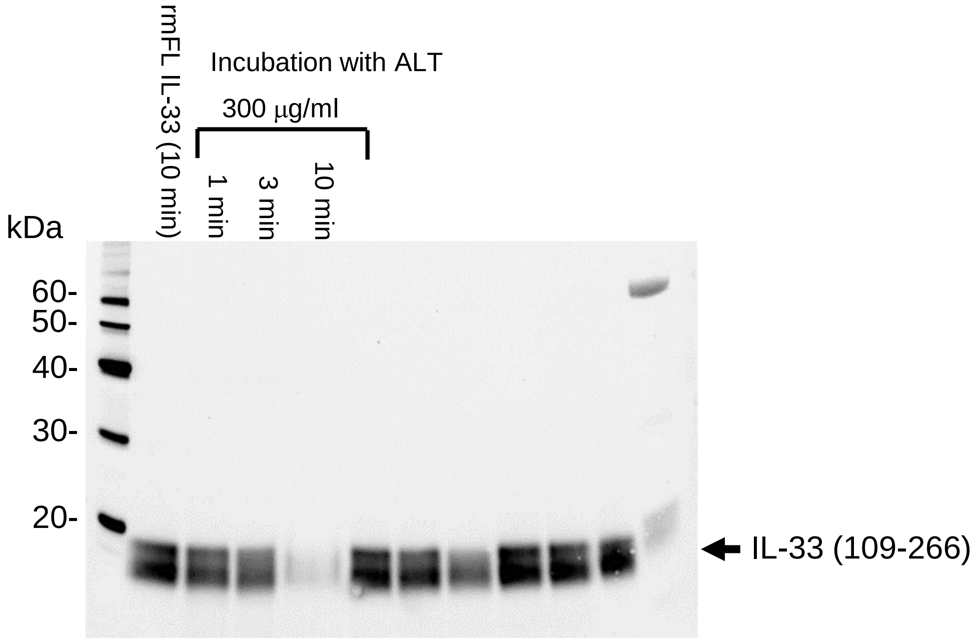

Fig. 6c
Lower panel

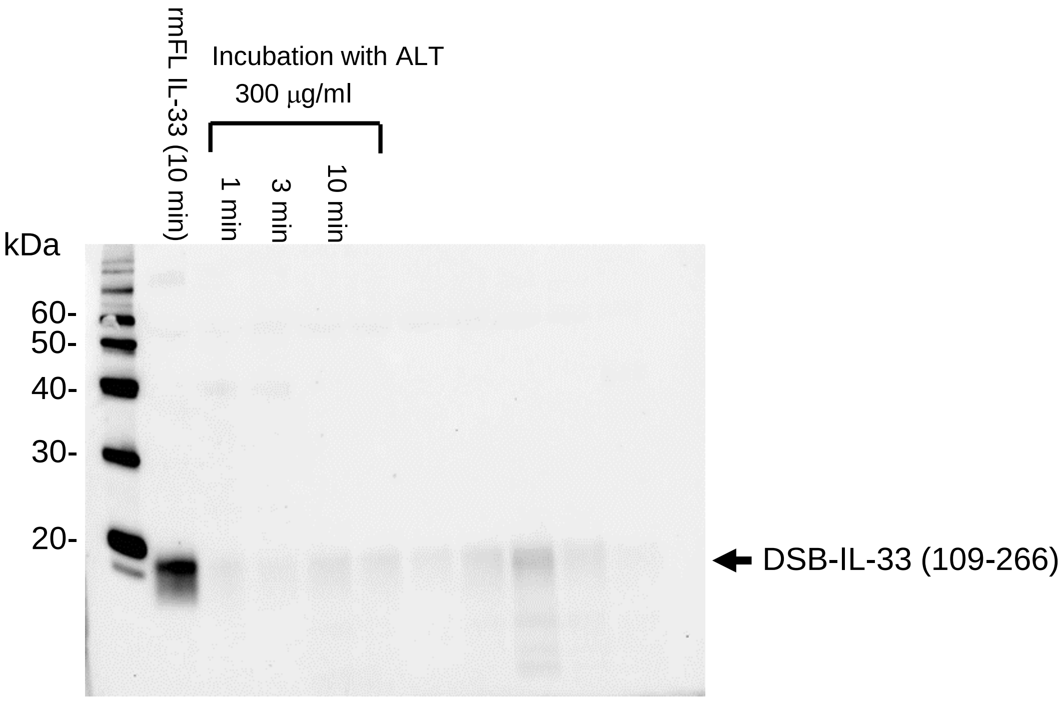

Supplementary Figure 13 – Full blots of Figure 5c

(**c**) Western blot of rmFL IL-33 lysate, rmIL-33 (109-266 aa) and DSB-rmIL-33 (109-266 aa) after incubation with 300 μg/ml ALT.

Supplementary figure 14

Fig. 5d
Upper panel

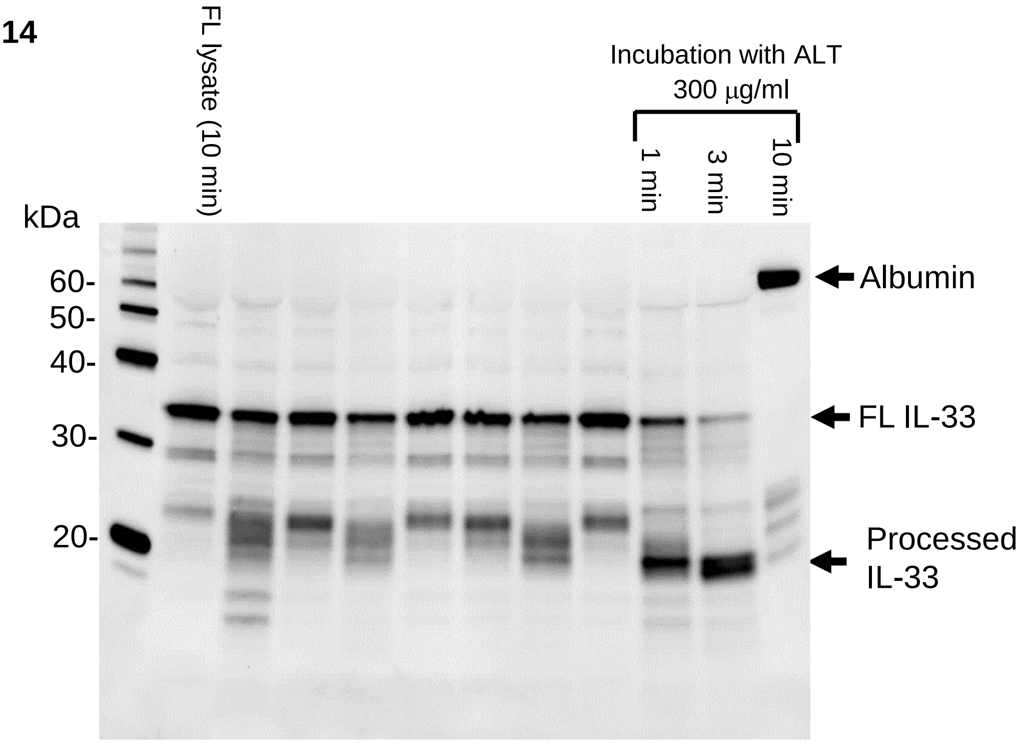

Fig. 5d
Middle panel

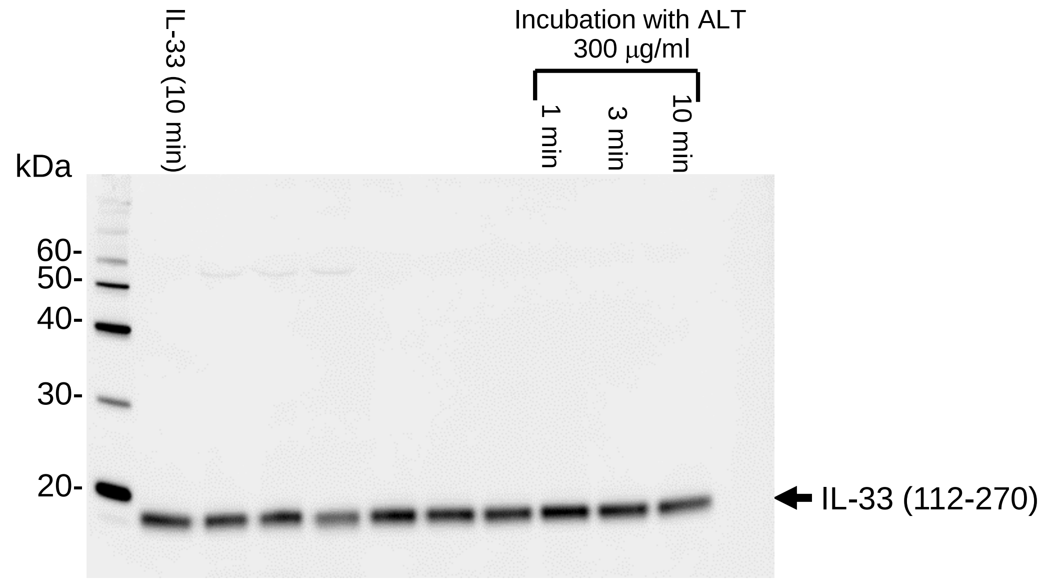

Fig. 5d
Lower panel

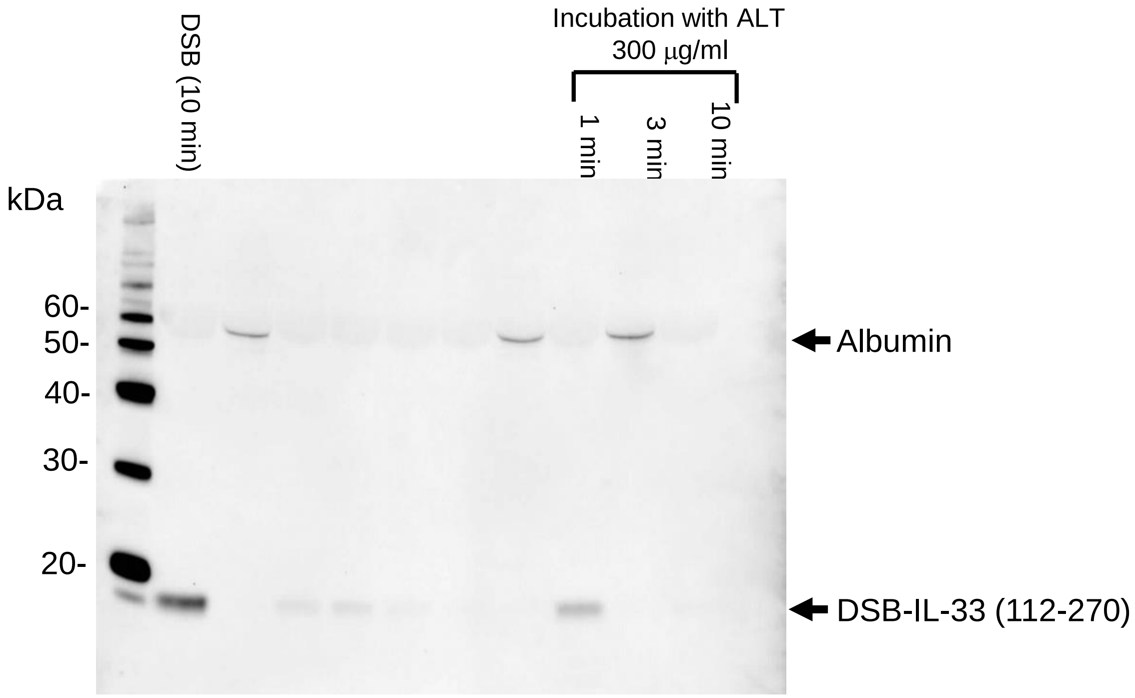

Supplementary Figure 14 – Full blots of Figure 5d

(**d**) Western blot of rhFL IL-33 lysate, rhIL-33 (112-270 aa), DSB-rmIL-33 (112-270 aa) before and after incubations with 300 μg/ml ALT for 1-10 min.

Supplementary figure 15

Fig. 5f

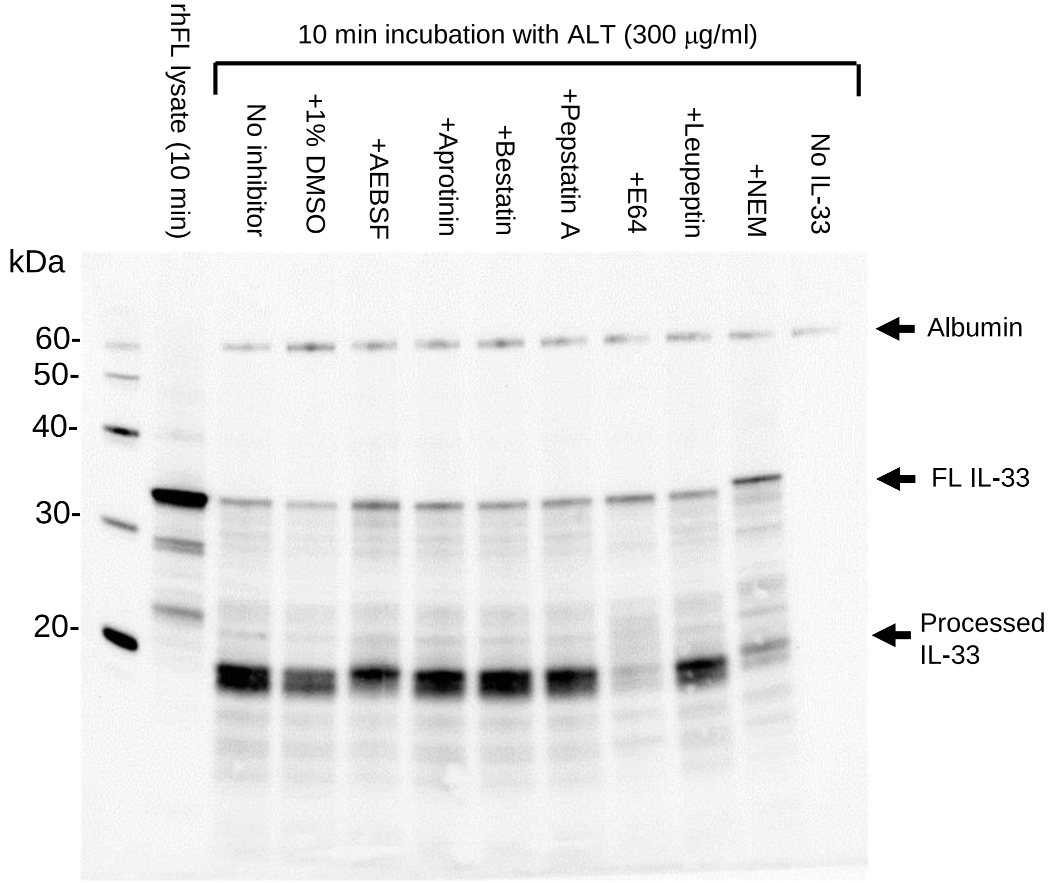

Fig. 5g

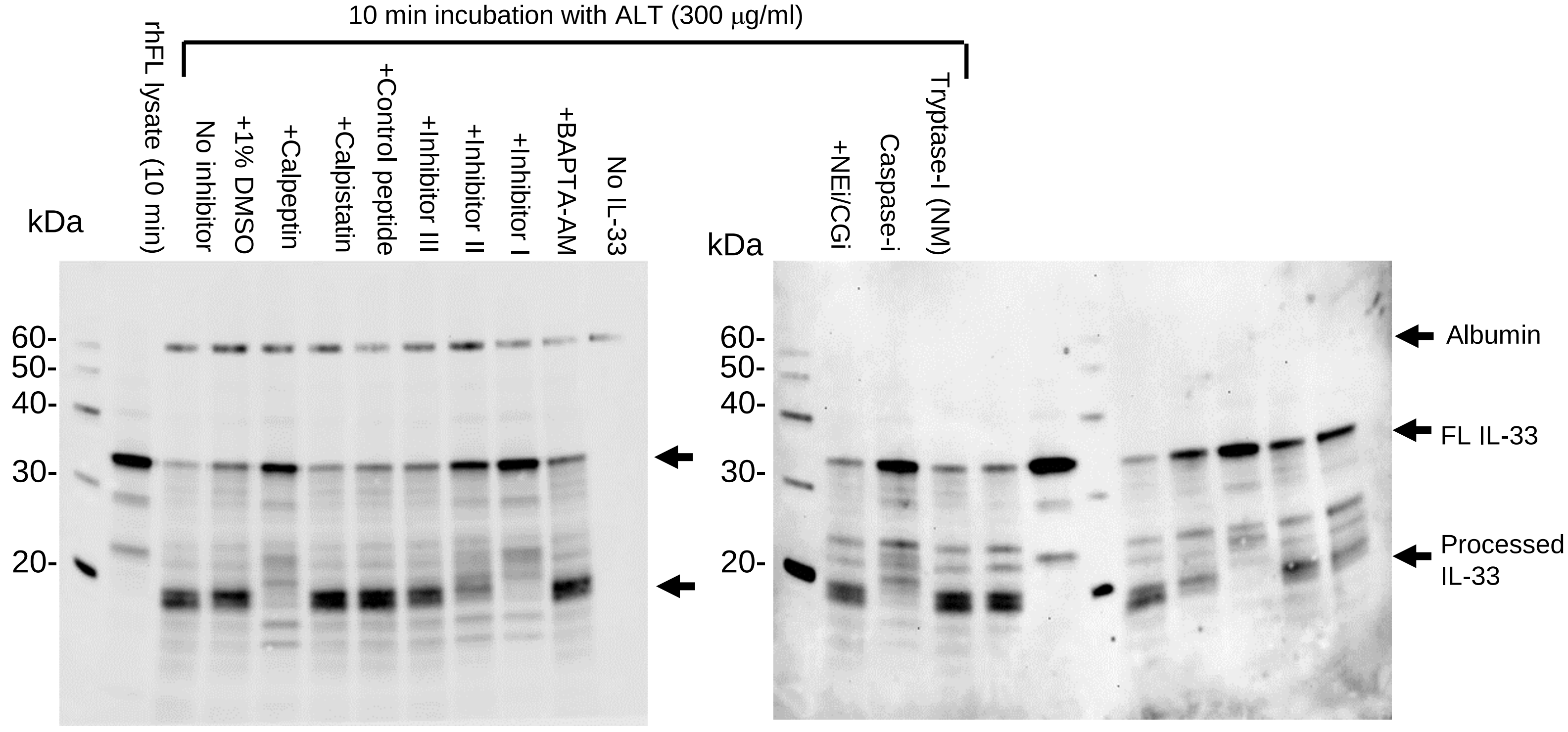

Supplementary Figure 15 – Full blots of Figure 5f and g

(**f**) Western blot of rhFL IL-33 lysate before and after incubation with 300 μg/ml ALT for 10 min. rhFL IL-33 was pre-incubated with protease inhibitors or BAPTA-AM, for 15 min prior to addition of ALT. Details of protease inhibitors are described in legend for Fig. 3c. (**g**) Western blot of rhFL IL-33 lysate with and without incubation with 300 μg/ml ALT or PBS for 10 min. Controls: FL lysate, lysate of HEK cells transfected with full length human IL-33. Details of protease inhibitors are described in legend for Fig. 3d.

Supplementary figure 16

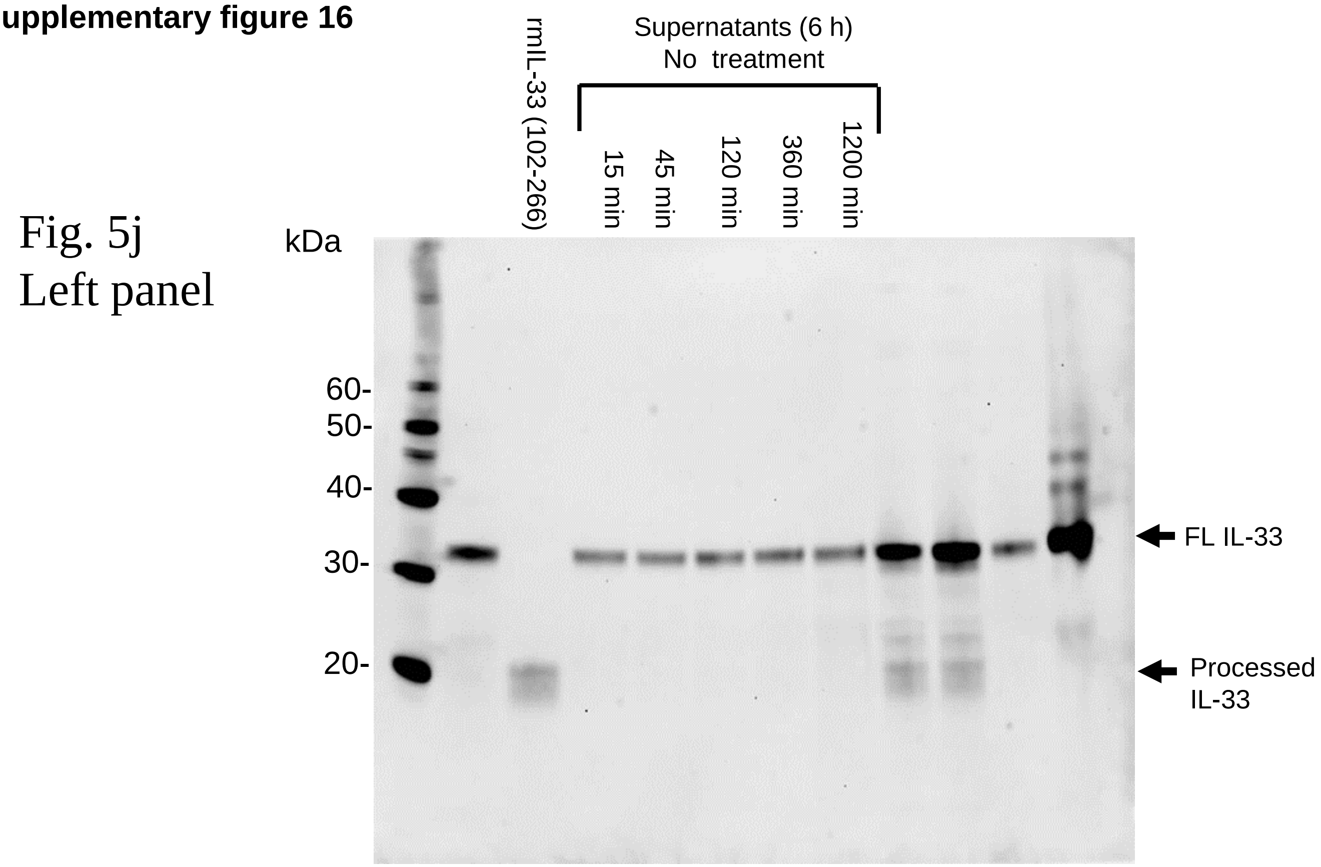

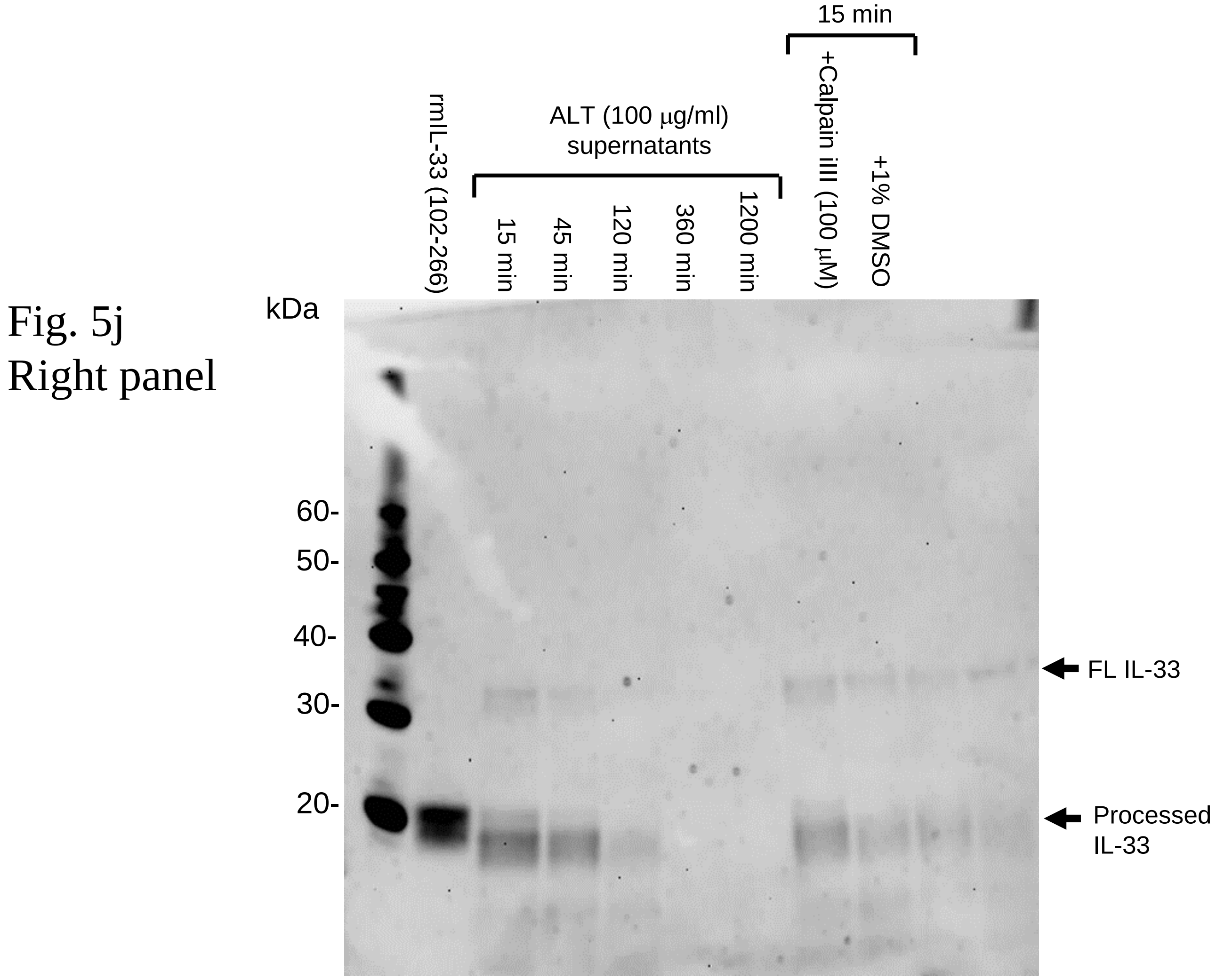

Supplementary Figure 16 – Full blots of Figure 5j

(**j**) Western blot of mouse CMT-64 cell supernatants 15-1200 min with (right panel) or without (left panel) 100 µg/ml ALT. ALT treatment was performed with or without pre-incubation for 15 min with 100 µg/ml calpain inhibitor III or 1% DMSO. Controls as Figure 3a.

Supplementary figure 17

Fig. 6b

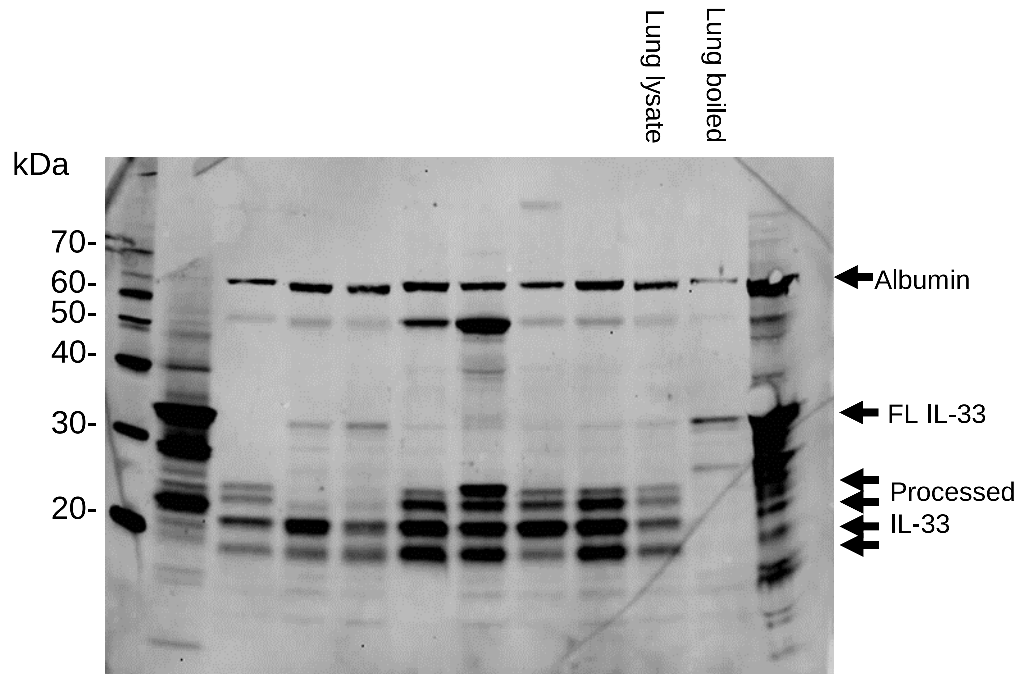

Fig. 6c

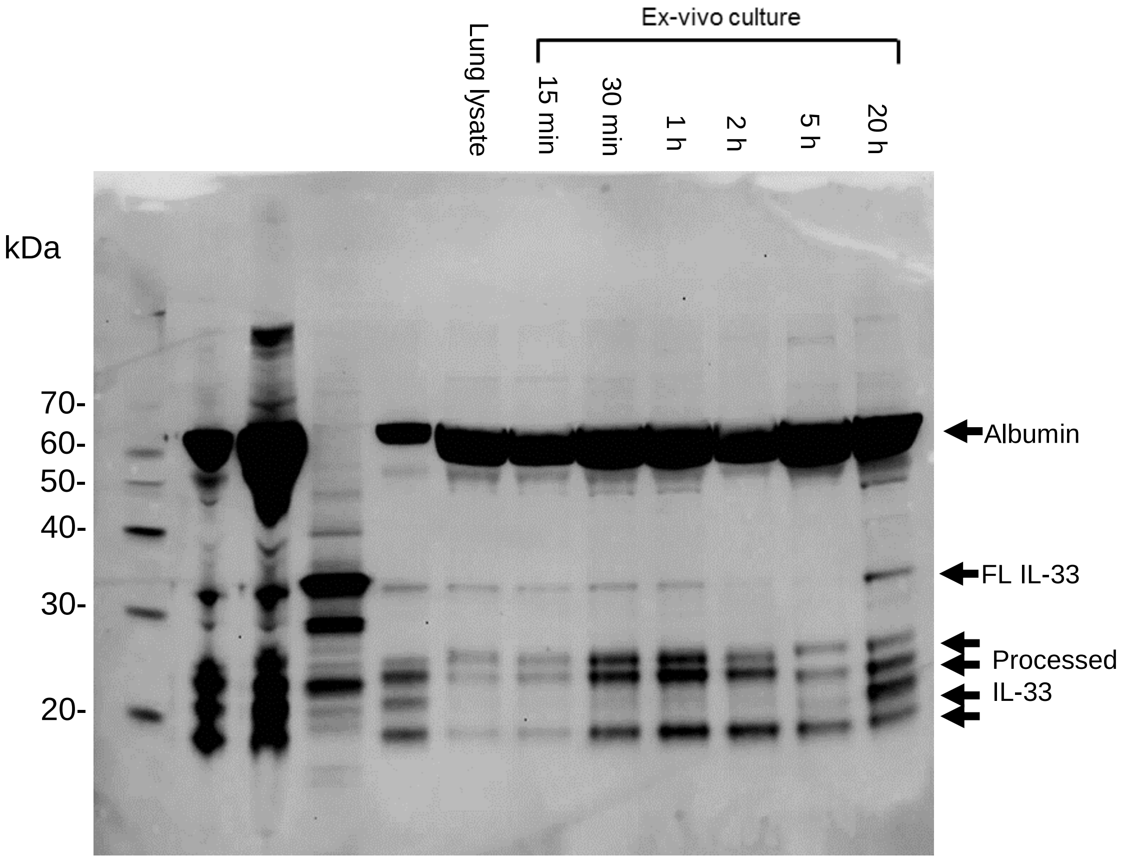

Supplementary Figure 17 – Full blots of Figure 6b and c

(**b**) Western blot of IL-33 expression in human COPD lung tissue (boiled in SDS-PAGE buffer) and lung tissue lysate. (**c**) Western blot of IL-33 expression in human COPD lung tissue and lung explant supernatants (15 min to 20 h after incubation).

Supplementary figure 18

Fig. 6e
Left panel

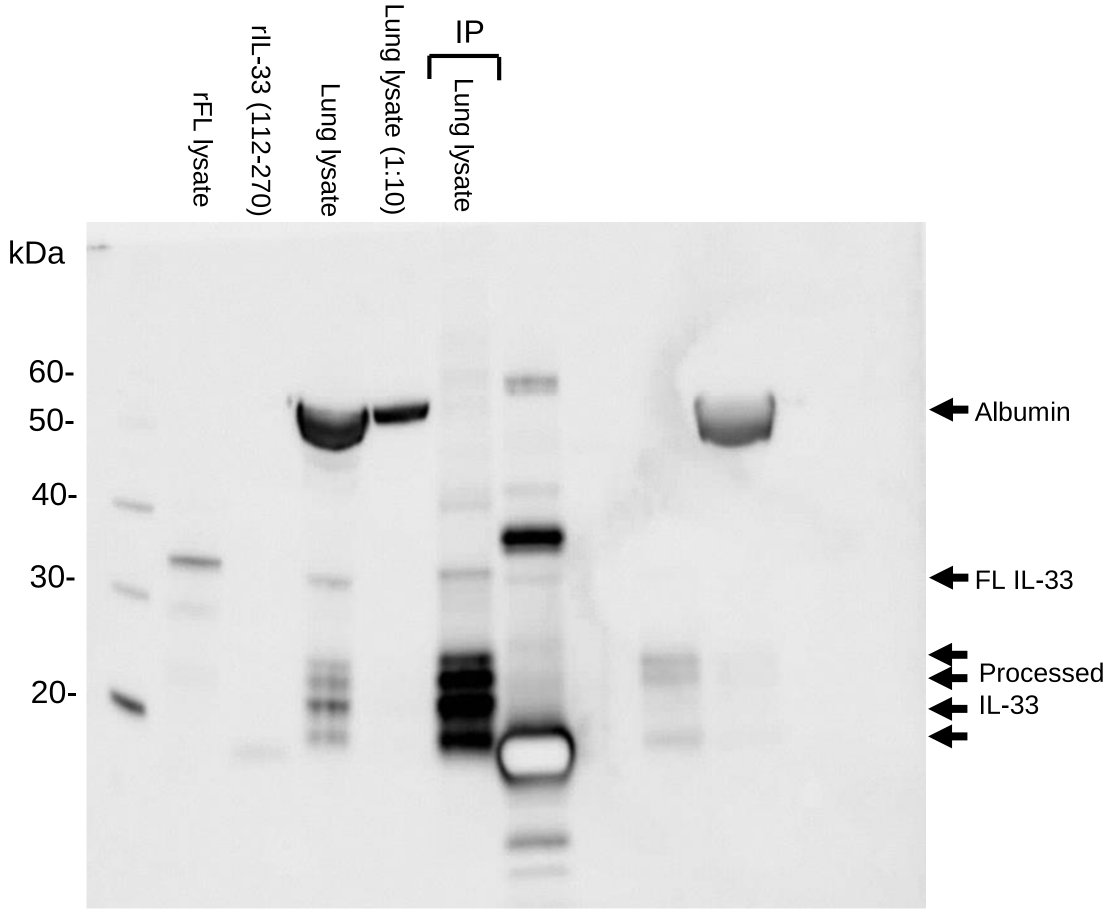

Fig. 6e
Right panel

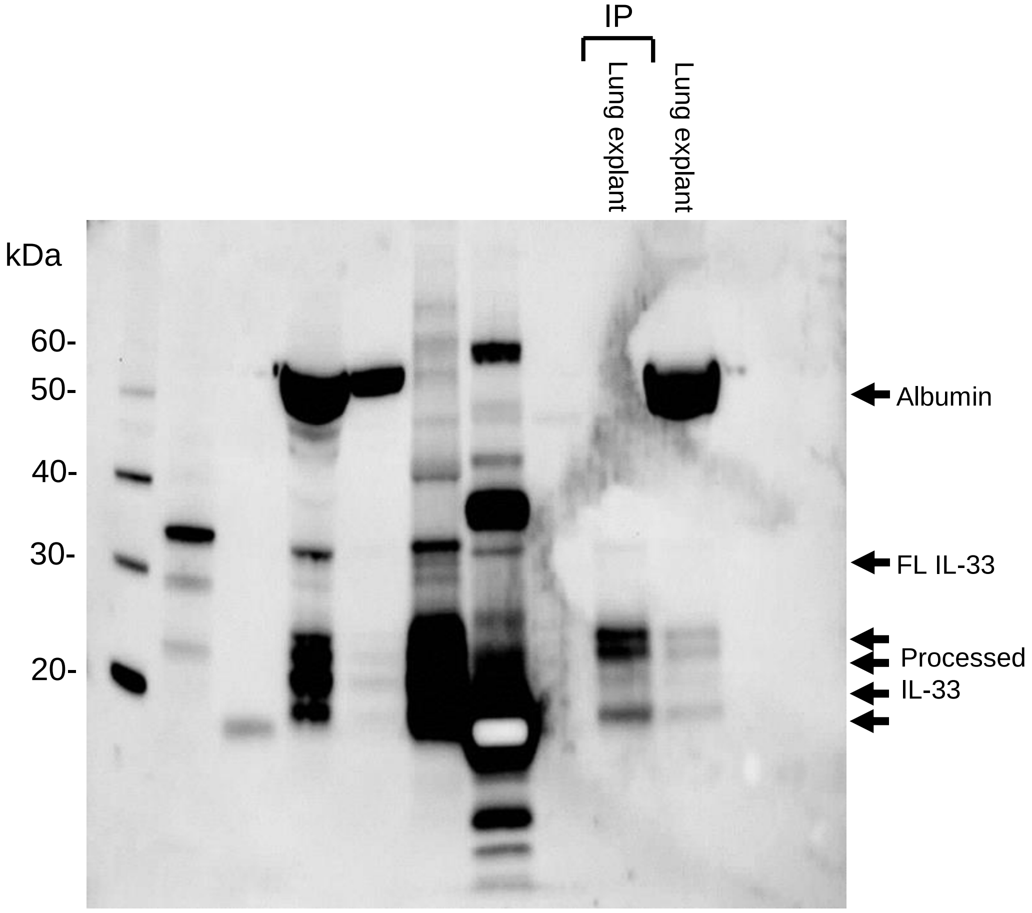

Supplementary Figure 18 – Full blots of Figure 6e

(**e**) Immunoprecipitation (IP) and western blot of IL-33 in human COPD lung lysate and explant supernatants (2 h). IL-33 was IP using anti-IL-33 mAb (#640050) and western blot performed with anti-IL-33 Ab (AF3625). Controls: rFL lysate, lysate of HEK cells transfected with full length human IL-33.

Supplementary figure 19

Fig. 6f

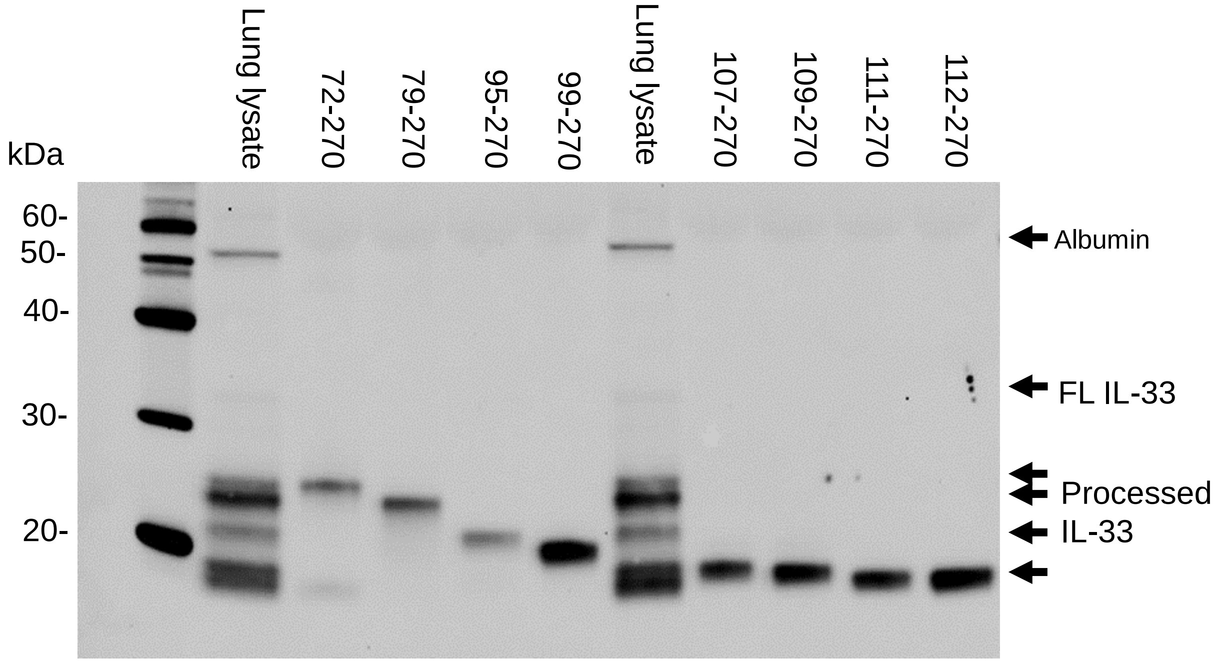

Fig. 6g

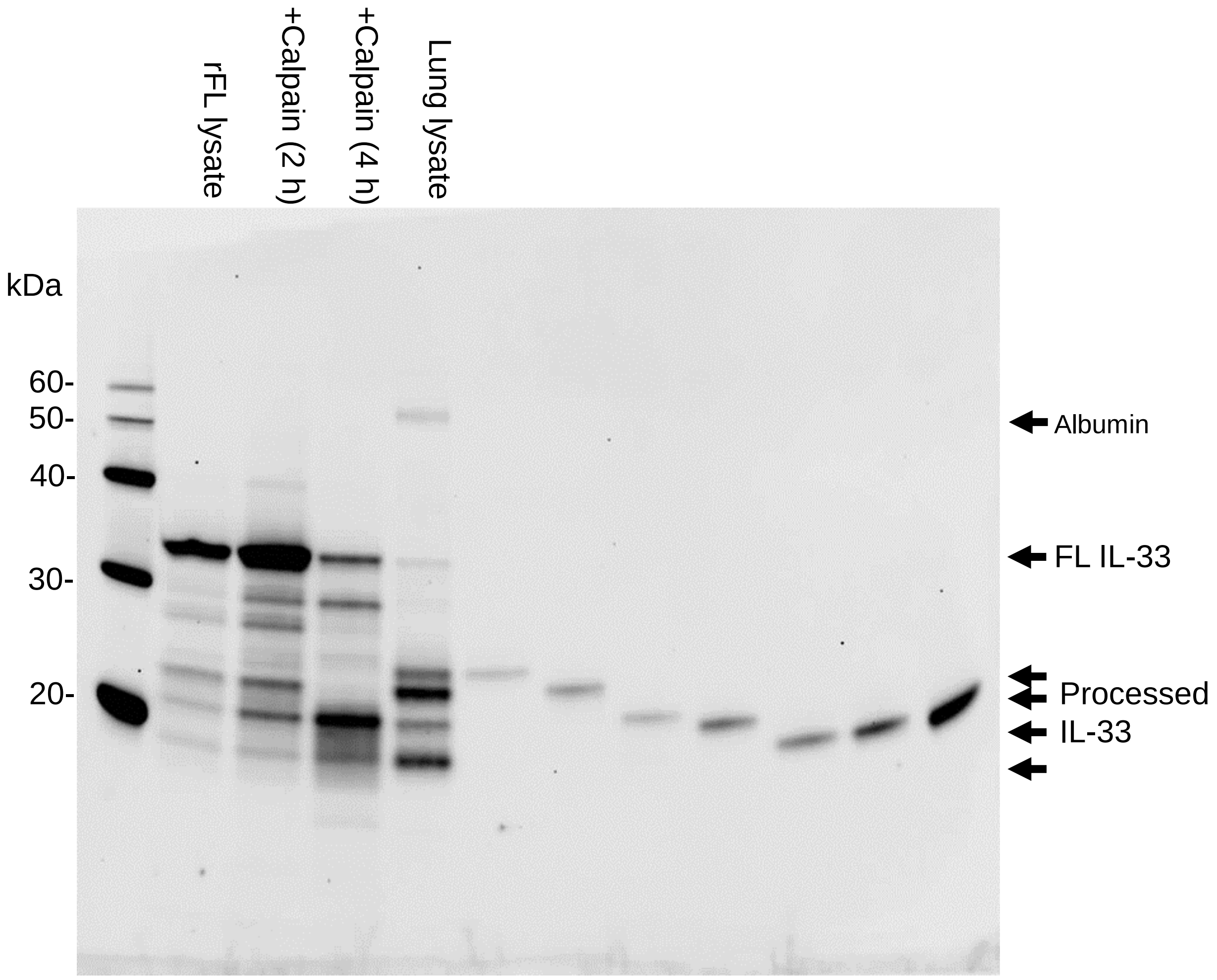

Supplementary Figure 19 – Full blots of Figure 6f and g

(**f**) Western blot of mature forms rhIL-33 (72-, 79-, 95-, 99-, 107-, 109-, 111-, 112-270 aa) and human lung lysate. (**g**) Western blot of rhFL IL-33 lysate, with or without incubation for 2-4 h with calpain, and human lung lysate. Controls as Fig. 6e.

Supplementary figure 20

Fig. 6h

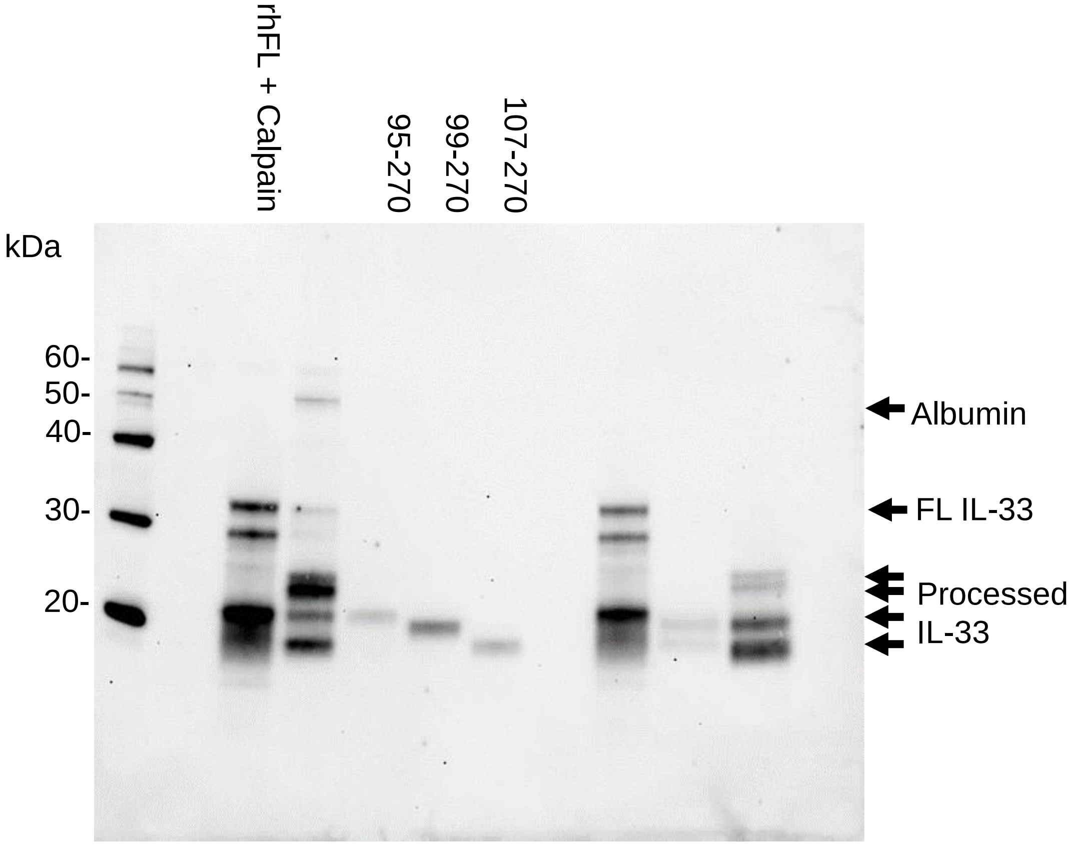

Supplementary Figure 20 – Full blots of Figure 6h and i

(**h**) Western blot of rhFL IL-33 lysate, incubated for 2 h with calpain, and mature forms of rhIL-33 (95-, 99-, 107-270 aa). (**i**) Western blot of rhFL IL-33 lysate, incubated for 10 min with ALT, and human lung lysate.

Supplementary Table 1

Human IL-33 form (aa)	Calculated MW (Da)	Protease/s
1-270	30760	
72-270	22450	Tryptase
79-270	21885	Tryptase
95-270	19826	Calpain<br>CG<br>Chymase
99-270	19438	NE
107-270	18518	Tryptase
109-270	18334	CG<br>Chymase
111-270	18081	Granzyme B
112-270	17994	

Supplementary Table 1:

Theoretical molecular weights (Da) of human IL-33 full length and processed form (72-, 79-, 95-, 99-, 107-, 109-, 111-270 aa) and recombinant mature IL-33 (112-270 aa). Abbreviations: aa, amino acids; CG, cathepsin G; NE, neutrophil elastase, MW, molecular weight.
